# Pathological extracellular matrix changes in decellularized normal appearing gray matter and subpial multiple sclerosis lesions

**DOI:** 10.1016/j.isci.2026.116020

**Published:** 2026-06-06

**Authors:** Jody M. de Jong, Justina C. Wolters, Marion H.C. Wijering, Joop de Vries, Susanne M. Kooistra, Bart J.L. Eggen, Wia Baron

**Affiliations:** 1 Department of Biomedical Sciences, University of Groningen, University Medical Center Groningen, Groningen, the Netherlands; 2 MS Center Noord Nederland, Groningen, the Netherlands; 3 Laboratory of Pediatrics, University of Groningen, University Medical Center Groningen, Groningen, the Netherlands; 4 Biomaterials & Biomedical Technology, University of Groningen, University Medical Center Groningen, Groningen, the Netherlands

**Keywords:** Clinical neuroscience, Health sciences, Medical specialty, Medicine, Neurology

## Abstract

Multiple sclerosis (MS) is a chronic CNS disease characterized by demyelinated lesions. Gray matter lesions (GMLs) are associated with neurodegeneration and clinical disability, and normal appearing gray matter (NAGM) also shows neuropathology. The extracellular matrix (ECM) supports neuronal health, with perineuronal nets (PNNs) regulating synaptic stability. Microglia remodel the ECM by secreting matrix metalloproteinases, while ECM composition affects microglia behavior. We examined ECM composition in decellularized human control gray matter, NAGM and GML slices and investigated its effects on microglia. Label-free quantitative mass spectrometry of decellularized control gray matter and NAGM revealed differential abundances in PNN ECM components and condition-specific synaptic and basement membrane ECM components. ECM composition was similar between decellularized GMLs and perilesional gray matter. Microglia introduced to decellularized NAGM and GML slices lost IBA1 expression in half the cells, while some remaining IBA1+ microglia acquired proinflammatory marker inducible nitric oxide synthase (iNOS). These findings suggest ECM alterations in NAGM influencing microglial characteristics potentially contributing to MS pathology.

## Highlights

• Significant changes in ECM composition in decellularized non-demyelinated MS gray matter
• Similar ECM composition between decellularized subpial lesions and perilesional gray matter
• Altered microglia characteristics upon introduction to decellularized subpial lesions

## Graphical abstract

**Figure:**
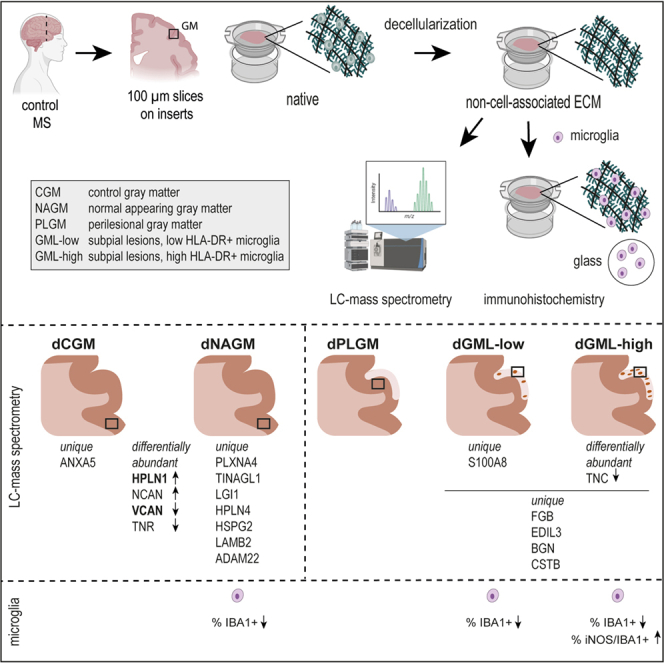

## Introduction

Multiple sclerosis (MS) is an inflammatory, demyelinating, and neurodegenerative disease of the central nervous system (CNS) that clinically presents as relapsing-remitting or progressive.^1^ In addition, across all disease courses, a more gradual and insidious accumulation of disability, not directly linked to acute relapses and known as PIRA (progression independent of relapse activity), has been recognized that is associated with neurodegeneration rather than inflammation.^2^ Unlike white matter lesions (WMLs), which are more typically linked to inflammatory demyelination, gray matter lesions (GMLs) often reflect ongoing neurodegenerative processes.^3^^,^^4^ In addition, GMLs are more strongly correlated with (progressive) physical disability^5^^,^^6^^,^^7^ than WMLs,^8^^,^^9^^,^^10^ particularly in primary progressive MS.^11^ When people with MS convert from a relapsing-remitting to a secondary progressive disease course, this shift is marked by accelerated neurodegeneration in gray matter.^5^ Additionally, GMLs are linked to cognitive deficits like memory impairment and attention deficits.^12^^,^^13^ Moreover, neurodegeneration can occur independently of demyelination.^14^^,^^15^^,^^16^^,^^17^ Neurodegenerative changes in normal appearing gray matter (NAGM) involve neuronal loss, axonal damage, and decreased synapse density, especially for inhibitory synapses, disrupting the balance between excitatory and inhibitory synapse activity.^18^^,^^19^^,^^20^ This indicates that neurodegenerative processes in NAGM and GMLs are key contributors to disease progression.

The extracellular matrix (ECM) plays an important role in maintaining neuronal health.^21^^,^^22^ In the adult brain, the ECM in gray matter is rich in non-fibrillar ECM components that are organized into specialized structures around neuronal cell bodies, at the myelin-free nodes of Ranvier and around and within synapse junctions (reviewed in Susuki et al.,^23^ Krishnaswamy et al.,^24^ Bruckner et al.,^25^ and Dauth et al.^26^). For example, perineuronal nets (PNNs) are net-like structures that surround the neuronal soma and proximal dendrites often extending to the axonal initial segment of predominantly parvalbumin-expressing interneurons.^27^^,^^28^ Composed of hyaluronan (HA), chondroitin sulfated proteoglycans (CSPGs), link proteins (HPLN1), and tenascins (TN), PNN function as a barrier, regulating synaptic stability by restricting synaptic plasticity and limiting the mobility of postsynaptic receptors.^29^^,^^30^^,^^31^ Functionally, PNNs play a crucial role in learning and memory^32^ and protect neurons from oxidative stress and activated microglia.^33^^,^^34^^,^^35^ By secreting ECM-degrading matrix metalloproteinases (MMPs), microglia modulate the composition and integrity of PNNs, leading to increased synaptic plasticity. If microglial MMP production becomes dysregulated, it may contribute to neurodegenerative processes.^36^^,^^37^ In the MS cortex, a subpopulation of microglia may initially remove pre-synapses from neuronal soma to prevent neuronal loss,^38^ highlighting the need for tight regulation of ECM remodeling.

While disturbed ECM remodeling impairs remyelination in WMLs by influencing the behavior of microglia and oligodendrocyte lineage cells,^39^^,^^40^ its role in gray matter MS pathology remains underexplored. To address this gap, we used decellularized tissue slices combined with discovery proteomics, to address differences in the non-cell-associated ECM composition (1) between gray matter tissue without apparent demyelination of control and MS donors and (2) between GMLs exhibiting different levels of inflammatory activity, as defined by low or high HLA-DR+ microglia numbers and non-demyelinated perilesional gray matter (PLGM). Furthermore, we explored how the non-cell-associated ECM affects microglia by introducing microglia to the topology-conserved decellularized slices.

## Results

### Several matrisome proteins are uniquely detected in decellularized NAGM

**Figure 1:**
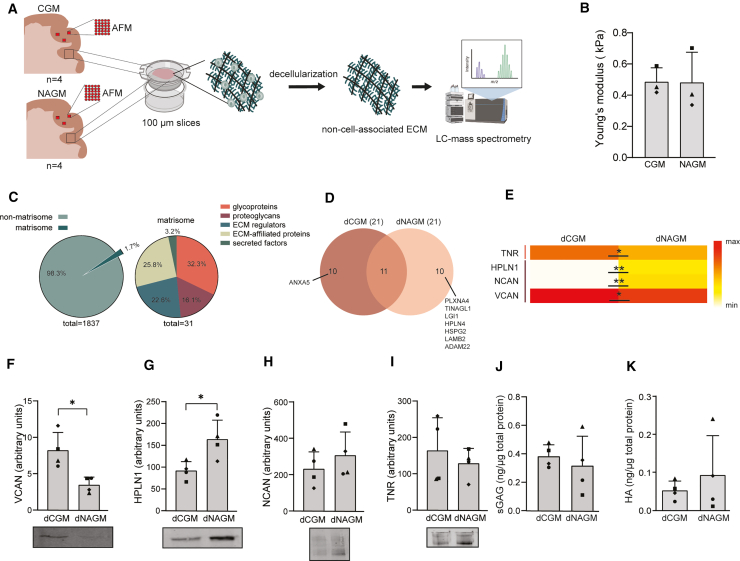
VCAN and HPLN1 are differentially abundant between decellularized CGM and NAGM (A) Experimental set-up. Control gray matter (CGM, *n* = 3–4, 4 non-demented donors) and normal appearing gray matter (NAGM, *n* = 3–4, 4 MS donors) from the superior temporal gyrus were selected based on the absence of detectable demyelination (PLP) and a low number of HLA-DR+ microglia. Snap-frozen tissues were sectioned in 100 μm slices and decellularized on inserts. Proteins in the decellularized tissue slices were identified with LC-mass spectrometry and analyzed with label-free quantitative proteomics. (B) Atomic force microscopy (AFM) stiffness measurements on unfixed 100 μm snap-frozen native CGM and NAGM slices. (C) Pie charts depicting the distribution of matrisome and non-matrisome proteins in decellularized CGM (dCGM) and NAGM (dNAGM) (left pie chart) and the distribution of different matrisome proteins over the indicated ECM-core and ECM-associated protein groups (right pie chart). (D) Venn diagram depicting the number of unique and common matrisome proteins in dCGM and dNAGM. In parentheses, the number of detected matrisome proteins present in at least one sample are indicated. Unique matrisome proteins found in at least two out of four samples are indicated. (E) Heatmap showing the relative abundance of differentially abundant matrisome proteins between dCGM and dNAGM. (F–H) Western blot quantification of VCAN (F), HPLN1 (G), NCAN (H), and TNR (I) levels in dCGM and dNAGM. (J and K) Quantification of ECM components sulfated glycosaminoglycans (sGAG, J) and hyaluronan (HA, K). Bars represent mean values. Error bars represent standard error of the mean. Symbols represent different donors (Table 1). Statistical analyses were performed using a Student’s *t* test to test for differences between dCGM and dNAGM (B, E–K, ∗*p* < 0.05, ∗∗*p* < 0.01).

To determine whether ECM in gray matter undergoes remodeling in the absence of detectable demyelination, we compared stiffness and ECM composition of gray matter brain tissue of control donors (CGM) to non-demyelinated gray matter brain tissue of MS donors (NAGM, Figure 1A). The thin and fragile nature of the tissue slices precluded rheology-based viscosity measurement. Therefore, to assess local mechanical properties of the tissue slices, we determined the Young’s modulus using atomic force microscopy (AFM), a nano-indentation method previously applied to quantify stiffness in brain tissue.^41^^,^^42^^,^^43^ AFM measurements on 100 μm native unfixed snap-frozen tissue revealed no significant differences in stiffness between CGM (0.49 ± 0.07 kPa) and NAGM (0.49 ± 0.16 kPa) (Figure 1B). In gray matter, non-cell-associated ECM, i.e., the network of proteins and macromolecules secreted by cells into the extracellular space provides structural support to neurons and elicit signals that influence cell behavior.^21^ To characterize non-cell-associated ECM composition of CGM and NAGM, we applied our recently optimized decellularization protocol for thin human gray matter brain slices.^44^ The decellularization procedure removed DNA (Figures S1A–S1C), corresponding to less than 1% residual DNA, comparable to decellularized tissues of other organs.^45^^,^^46^ The reduction of intracellular proteins, such as actin and histone H3 (Figures S1D–S1G), and preservation of ECM components laminin, HA, and sulfated glycosaminoglycans (sGAGs, Figures S1A, S1H–S1K), further supports effective decellularization. Reliable stiffness measurements for decellularized gray matter tissue were not possible due to probe entrapment in soft tissue. With liquid chromatography mass spectrometry of decellularized CGM (dCGM) and NAGM (dNAGM) a total of 1,837 proteins were detected, corresponding to overrepresentation of Gene Ontology (GO) terms that collectively refer to a role in the regulation and functioning of neurons, including processes involved in neuronal structure and synaptic organization (Figure S2A). Of the 1,837 total proteins, 31 belong to a computationally assembled ECM proteome, termed the matrisome,^47^ consisting of ECM-core and ECM-associated proteins (Figure 1C). For a more precise understanding on how alterations in matrisome proteins may contribute to disease pathology, we focused our further analysis on these matrisome proteins in two complementary ways. First by identifying uniquely detected proteins within one tissue group (defined as being present in two out of four samples), and second, by identifying matrisome proteins differentially abundant between the tissue groups (Tables S1 and S2). Of the 31 matrisome proteins, annexin-5 (ANXA5) was not present in any dNAGM but detected in dCGM. In dNAGM, seven proteins were uniquely detected in dNAGM that were not present in dCGM (Figure 1D; Tables S1 and S2). More specifically, plexin A4 (PLXNA4) and tubulointerstitial nephritis antigen like 1 (TINAGL1) were uniquely detected in all four dNAGM, and leucine-rich glioma inactivated 1 (LGI1) and HA and proteoglycan link protein 4 (HPLN4) were detected in three dNAGM. Heparin sulfate proteoglycan 2 (HSPG2, also known as perlecan), laminin subunit B2 (LAMB2), and disintegrin and metalloproteinase domain-containing protein 22 (ADAM22) were found in two out of four dNAGM. The identification of these matrisome proteins exclusively in either dCGM or dNAGM indicates alterations in ECM composition occurring in the absence of detectable demyelinating pathology.

**Table 1:** Donor information

	Tissue type^a^	Symbol	MS type^b^	sex	f:m^c^	age	median±SD^d^	pmd^e^ (h:m)	median±SD^d^	disease duration (years)	median±SD^d^	cause of death
Proteomics	CGM^g^		–	f	2:2	60	66.0 ± 7.2	08:25	6:57 ± 2:19	–	–	euthanasia
	CGM^g^		–	m	–	73	–	09:10	–	–	–	cardiac arrest
	CGM^g^		–	f	–	60	–	05:30	–	–	–	euthanasia
	CGM		–	m	–	72	–	04:20	–	–	–	cardiogenic and distributive shock
	NAGM^g^		SPMS	m	2:2	50	58.5 ± 13.8	10:50	9:43 ± 1.34	21	25.5 ± 2.6	euthanasia
	NAGM^f^^,^^g^		SPMS	f	–	76	–	07:55	–	25	–	euthanasia, long cancer with brain metastases
	NAGM^f^		SPMS	m	–	67	–	11:00	–	26	–	sudden death with ms
	NAGM^f^^,^^g^		SPMS	f	–	47	–	08:35	–	27	–	aspiration pneumonia
	GML-low + PLGM		SPMS	f	3:1	48	56.0 ± 11.9	05:50	8:10 ± 1:34	22	27.8 ± 4.4	congestive heart failure
	GML-low		SPMS	m	–	56	–	09:35	–	32	–	end stage MS with urosepsis
	GML-low		SPMS	f	–	56	–	08:25	–	32	–	respiratory insufficiency by pneumonia
	GML-low^f^		SPMS	f	–	76	–	07:55	–	25	–	euthanasia, long cancer with brain metastases
	GML-high + PLGM		PPMS	f	2:2	73	65.50 ± 11.2	07:05	8:02 ± 1:46	30	28.5 ± 4.0	euthanasia
	GML-high		SPMS	m	–	64	–	07:30	–	35	–	euthanasia
	GML-high + PLGM^f^		SPMS	f	–	47	–	08:35	–	27	–	aspiration pneumonia
	GML-high + PLGM^f^		SPMS	m	–	67	–	11:00	–	26	–	sudden death with MS
Recellularization	CGM		–	f	1:2	60	72.0 ± 10.5	08:25	5:30 ± 2:10	–	–	euthanasia
	CGM		–	m	–	72	–	04:20	–	–	–	cardiogenic and distributive shock
	CGM		–	m	–	81	–	05:30	–	–	–	metastasis prostate carcinoma
	NAGM		SPMS	f	2:1	52	52.0 ± 10.4	9:35	9:35 ± 1:13	16	26.0 ± 6.1	pneumonia
	NAGM		SPMS	m	–	67	–	11:00	–	26	–	sudden death with MS
	NAGM		SPMS	f	–	47	–	08:35	–	27	–	aspiration pneumonia
	GML-low		SPMS	f	1:2	48	56.0 ± 15.5	05:50	8:50 ± 1:59	22	31.3 ± 7.4	congestive heart failure
	GML-low		SPMS	m	–	56	–	09:35	–	32	–	end stage MS with urosepsis
	GML-low		SPMS	m	–	78	–	08:50	–	40	–	MS, dehydration and cachexia
	GML-high		SPMS	m	2:2	64	65.5 ± 11.2	07:30	8:02 ± 1:46	35	28.5 ± 4.0	euthanasia
	GML-high		PPMS	f	–	73	–	07:05	–	30	–	euthanasia
	GML-high		SPMS	f	–	47	–	08:35	–	27	–	aspiration pneumonia

No significant differences in age, pmd and disease duration between different groups (proteomics: Student’s *t* test to compare CGM and NAGM, one-way ANOVA to compare GML-low, GML-high, and PLGM; recellularization: one-way ANOVA to compare CGM, NAGM, GML-low, and GML-high).

a CGM, control gray matter; NAGM, normal appearing gray matter; GML–low, subpial gray matter lesion with a low density of HLA-DR+ cells; GML–high, subpial gray matter lesion with a high density of HLA-DR+ cells; PLGM, perilesional gray matter; CGM and NAGM are obtained from superior temporal gyrus.

b PPMS = primary progressive MS; SPMS = secondary progressive MS.

c :m, female:male ratio.

d median ± standard deviation.

e pmd = postmortem delay in h:m = hours:minutes;

f same donors for NAGM and GML.

g tissue also used for atomic force microscopy.

### VCAN and HPLN1 are differentially abundant between decellularized CGM and NAGM

In addition to the uniquely identified proteins in either dCGM or dNAGM, we determined differences in ECM composition between dCGM and dNAGM by comparing the abundances of matrisome proteins in both groups. Of the 31 matrisome proteins, nine proteins were present in both dCGM and dNAGM in at least two samples (Figure 1D; Tables S1 and S2) of which four proteins were differentially abundant between dCGM and dNAGM (Figure 1E). Tenascin-R (TNR) and versican (VCAN) were less abundant in dNAGM, whereas HA and proteoglycan link protein 1 (HPLN1) and neurocan (NCAN) were enriched in dNAGM (Figure 1E). Aggrecan (ACAN), brevican (BCAN), LGI3, ADAM23, and HPLN2 were equally abundant in dCGM and dNAWM. Western blot analysis confirmed lower VCAN levels and higher HPLN1 levels in dNAGM (Figures 1F and 1G). The differences in TNR and NCAN abundancy identified in the proteomic analysis were not evident with western blot analysis (Figures 1H and 1I), likely attributable to differences in antibody epitope recognition versus mass spectrometry peptide coverage. Four of the nine common detected matrisome proteins were proteoglycans. Proteoglycans consist of a core protein to which one or more glycosaminoglycan (GAG) chains are covalently attached. Quantitative analysis of the amount of sulfated GAGs (sGAG) and HA, as non-sulfated GAG mostly produced by neurons, showed similar sGAG and HA levels between dCGM and dNAGM (Figures 1J and 1K). Hence, while sGAG and HA levels are comparable between dCGM and dNAGM, VCAN and HPLN1 are differentially abundant between decellularized CGM and NAGM. VCAN is enriched in decellularized CGM and HPLN1 in decellularized NAGM. As ECM remodeling often occurs in response to injury, changes in cortical non-cell-associated ECM upon demyelinating injury were examined next.

### NCAN and HPLN1 are less abundant in decellularized demyelinated mouse cortex

**Figure 2:**
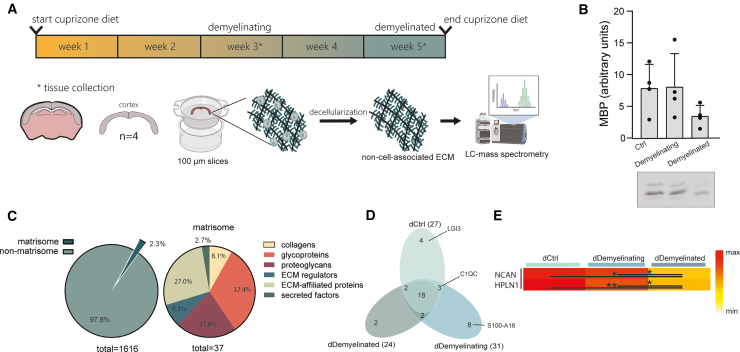
NCAN and HPLN1 are less abundant in decellularized demyelinated mouse cortex (A) Experimental set-up. Mice were fed with normal chow (*n* = 4) or with a cuprizone diet for 3 weeks (demyelinat*ing*, *n* = 4) or 5 weeks (demyelinat*ed*, *n* = 4). Snap-frozen cortices were sectioned in 100 μm slices and decellularized on inserts. Proteins in the decellularized tissue slices were identified with LC-mass spectrometry and analyzed with label free quantitative proteomics. (B) Western blot quantification of MBP levels in ctrl, demyelinating, and demyelinated cortices. (C) Pie charts depicting the distribution of matrisome and non-matrisome proteins in decellularized control (dCtrl), demyelinating (dDemyelinating), and demyelinated (dDemyelinated) cortex (left pie chart) and the distribution of different matrisome proteins over the indicated ECM-core and ECM-associated groups (right pie chart). (D) Venn diagram depicting the number of unique and common matrisome proteins in dCtrl, dDemyelinating, and dDemyelinated. In parentheses, the number of detected matrisome proteins present in at least one sample are indicated. Unique matrisome proteins in at least two out of four samples or in one group or in at least one sample of two groups are indicated. (E) Heatmap showing the relative abundance of the differentially abundant matrisome proteins. Bars represent mean values. Error bars represent standard error of the mean. Dots represent different mice. Statistical analyses were performed using a one-way ANOVA with Tukey’s multiple comparisons test (B, not significant) or a Student’s *t* test to test for differentially abundant matrisome proteins between dCtrl and dDemyelinating, dCtrl and dDemyelinated, and dDemyelinating and dDemyelinated (E, ∗*p* < 0.05).

To study whether the differential abundance of VCAN and HPLN1 may be a sign of early demyelination, we next analyzed the ECM composition in the cortex upon cuprizone-induced demyelination (Figure 2A). To induce demyelination, mice were fed with cuprizone for 3 (demyelinat*ing*) or 5 weeks (demyelinat*ed*). Demyelination was confirmed by reduced myelin basic protein (MBP) protein levels (Figure 2B). ECM was extracted from 100 μm slices of the cortex by a decellularization protocol optimized for mouse brain tissue (Figure S3). LC-mass spectrometry identified 1,616 proteins, associated with GO terms describing processes related to energy metabolism, cytoskeletal dynamics, and neuronal signaling (Figure S2B). Among these proteins, 31 matrisome proteins were detected, including collagen, glycoproteins, proteoglycans, ECM regulators, ECM-affiliated proteins and secreted factors (Figures 2C and S2B; Table S2). LGI3 was uniquely detected in decellularized control cortices (dCtrl), and S100A16 was only detected in decellularized cortices after 3 weeks cuprizone feeding (dDemyelinating, Figure 2D; Tables S3 and S4). C1QC was detected in dCtrl and dDemyelinating cortices and not in decellularized cortices after 5 weeks cuprizone feeding (dDemyelinated) (Figure 2D; Tables S3 and S4). No matrisome proteins were uniquely detected in dDemyelinated cortices. Of the 13 matrisome proteins found in at least two decellularized slices in all three tissue groups, NCAN and HPLN1 were less abundant in dDemyelinated compared to dCtrl and dDemyelinating cortices. VCAN and TNR were similarly abundant in dCtrl, dDemyelinating, and dDemyelinated cortices (Tables S3 and S4). To assess whether the lower abundance of NCAN and HPLN1 was transient, we compared LC-mass spectrometry analyses of decellularized cortices after 5 weeks cuprizone feeding (dDemyelinated) with decellularized cortices from mice that were subjected to 5 weeks cuprizone feeding followed by either 1-week normal chow (dRemyelinat*ing*) or 2 weeks normal chow (dRemyelinat*ed*) (Figure S4A). Across the conditions, a total of 1,408 proteins were identified of which 37 were classified as matrisome proteins (Figure S4B; Tables S5 and S6). No matrisome proteins were uniquely detected in at least two samples of each condition (Figure S4C). Among the matrisome proteins detected in the decellularized cortices, NCAN was more abundant in dRemyelinated compared to dDemyelinated cortices (Figure S4D; Tables S5 and S6). HPLN1 was also more enriched in dRemyelinated cortices, which did not reach statistical significance (Tables S5 and S6). These findings indicate that cuprizone-mediated demyelination in the cortex is associated with a transient loss of NCAN and HPLN1 at demyelinated conditions in the non-cell-associated ECM.

### TNC is less abundant in decellularized subpial GMLs with high HLA-DR+ microglia presence

**Figure 3:**
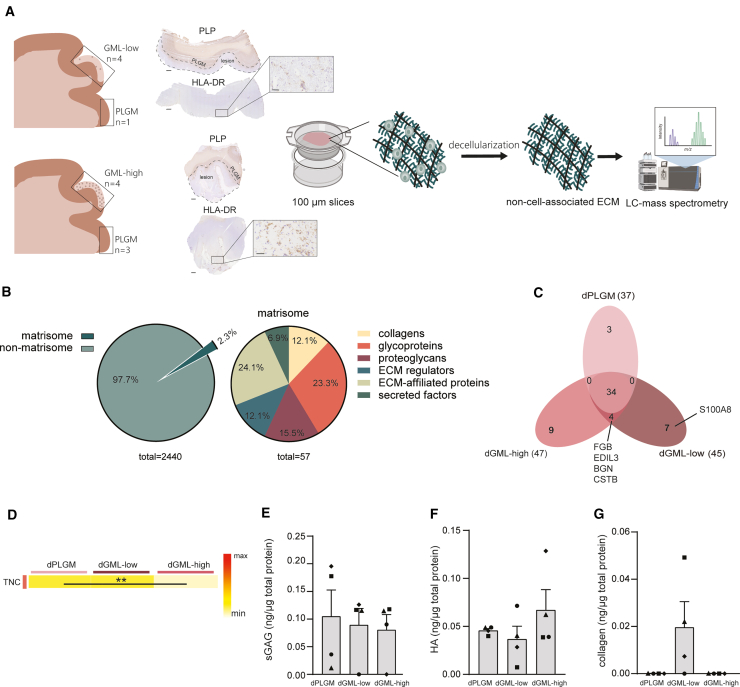
TNC is less abundant in decellularized subpial GMLs with high HLA-DR+ microglia presence (A) Experimental set-up. Gray matter lesions (GMLs, *n* = 8, 8 donors) and surrounding perilesional gray matter (PLGM, *n* = 4, 4 donors) were selected based on the absence or presence of myelin (PLP) and HLA-DR+ cells (mainly microglia). GMLs are divided in GMLs with low (GML-low, *n* = 4) or high (GML-high, *n* = 4) HLA-DR+ microglia numbers. Snap-frozen tissues were sectioned in 100 μm slices and decellularized on inserts. Proteins in the decellularized tissue slices were identified with LC-mass spectrometry and analyzed with label free quantitative proteomics. Representative images of PLP and HLA-DR are shown. Scale bars are 1 mm (overview) and 50 μm (inset). (B) Pie charts depicting the distribution of matrisome and non-matrisome proteins in mass spectrometry analysis of dPLGM, dGML-low, and dGML-high (left pie chart) and the distribution of different matrisome proteins over the indicated ECM-core and ECM-associated protein groups (right pie chart). (C) Venn diagram depicting the number of unique and common matrisome proteins in dPLGM, dGML-low, and dGML-high. In parentheses, the number of detected matrisome proteins present in at least one sample are indicated. Unique matrisome proteins in at least two out of four samples or in one group or in at least one sample of two groups are indicated. (D) Heatmap showing the relative abundance of the differentially abundant matrisome proteins between dPLGM, dGML-high and dGML-low. (E–G) Quantification of ECM components sulfated glycosaminoglycans (sGAG, E), hyaluronan (HA, F), and collagen (G). Bars represent mean values. Error bars represent standard error of the mean. Symbols represent different donors (Table 1). Statistical analyses were performed using a Student’s *t* test to test for differences between dPLGM and dGML-low, dPLGM and dGML-high, and dGML-low and dGML-high (D, ∗∗*p* < 0.01) or one-way ANOVA with Tukey’s multiple comparisons test (E–G, not significant).

To determine whether similar ECM remodeling occurs in demyelinated areas of the human MS cortex and the cuprizone-induced demyelinated mice cortex, we characterized the ECM composition in decellularized subpial GMLs, taking into account the role of microglia in ECM remodeling. Subpial GMLs were stratified based on the density of HLA-DR+ cells (mainly microglia in gray matter (GM)) into lesions with high (GML-high) or low (GML-low) microglia presence. In addition, surrounding non-demyelinated PLGM was included. Thin 100 μm tissue slices of these human tissues and areas were decellularized and subjected to proteomics analyses to identify ECM protein changes (Figure 3A). A total of 2,440 proteins were detected, predominantly associated with GO terms indicating modulation of neuronal connectivity and function, involving biological processes related to neuronal structure organization, intracellular transport, and synaptic regulation (Figure S2C). Of these proteins, 57 were matrisome proteins (Figure 3B; Tables S7 and S8). When their presence in at least two samples per tissue group and absence in the other conditions were considered, no matrisome proteins were uniquely detected in decellularized PLGM (dPLGM) and GML-high (dGML-high), while S100A8 was only found in decellularized GML-low (dGLM-low, Figure 3C; Tables S7 and S8). Notably, EGF-like repeats and discoidin domains 3 (EDIL3), the beta component of fibrinogen (FGB), biglycan 1 (BGN), and cystatin B (CSTB) were detected in at least one sample of both dGMLs, while not found in dPLGM (Figure 3C; Tables S7 and S8). Of the 19 matrisome proteins that were present in at least two of the four decellularized samples of all three conditions, tenascin C (TNC) was differentially abundant, being significantly less abundant in dGML-high compared to dPLGM (Figure 3D; Tables S7 and S8). Notably, in the cuprizone model, TNC was uniquely detected in one dDemyelinating sample (Tables S3 and S4). TNC was not detectable with western blot, likely, due to overall low expression levels in decellularized tissue slices. NCAN and HPLN1, ECM-core proteins that were less abundant in dDemyelinated upon cuprizone-induced demyelination, were equally abundant in dGMLs and dPLGM (Tables S7 and S8). Furthermore, sGAG and HA levels were similar between dGMLs and dPLGM (Figures 3E and 3F). In contrast to dCGM and dNAGM (Figure 1C; Tables S1 and S2), collagens were detected in the proteomic profiles of dPLGM, dGML-low, and dGML-high (Figure 3B; Tables S7 and S8). Quantitative analyses of total collagen levels revealed that collagen was present in three out of four dGML-low, whereas it was undetectable in dCtrl and dGML-high (Figure 3G). Hence, proteomic profiling of dGMLs identified five matrisome proteins uniquely present in dGMLs, while TNC was less abundant in dGMLs with high HLA-DR+ microglia presence compared to dPLGM.

### Exposure to MS-relevant factors does not reduce TNC expression in hiPSC-derived astrocytes

**Figure 4:**
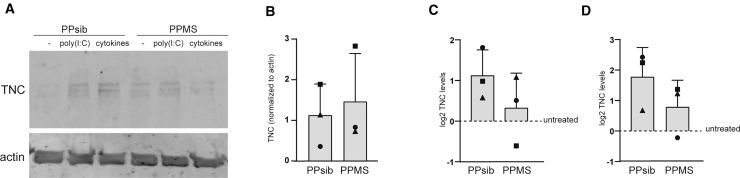
Exposure to MS-relevant factors do not reduce TNC expression in hiPSC-derived astrocytes (A) Representative blots depicting tenascin-C (TNC) expression of hiPSC-derived astrocytes from people with PPMS (PPMS-iAstro, *n* = 3, 3 donors) and their non-affected siblings (PPsib-iAstro, *n* = 3, 3 donors) when left untreated (−) or treated with poly(I:C) or a mix of pro-inflammatory cytokines (TNF-α, IFN-γ, IL1-β). (B–D) Western blot quantification of TNC expression in untreated (B), poly(I:C)-treated (C), and cytokine-treated (D) iAstro. Bars represent the mean normalized to actin (B), or the mean of relative levels to its untreated condition (log2fold, C and D), which was set to 1 in each independent experiment (horizontal line). Error bars represent standard of error of the mean. PPMS donors and corresponding sibling are indicated with the same symbol. Statistical analyses were performed using a Student’s *t* test (B, not significant) or one-sample *t* test (C,D, not significant) to test for differences between groups or treatments, respectively.

As TNC is mainly produced and secreted by astrocytes,^48^ we next questioned whether astrocytic TNC expression and deposition were diminished by factors present in GMLs. hiPSC-derived astrocytes (iAstro) represent a suitable model to study injury-naive astrocytes and to assess the effect of defined environmental factors relevant to MS lesions. Accordingly, hiPSC-derived astrocytes from donors with PPMS (PPMS-iAstro) and their non-affected siblings (PPsib-iAstro) were exposed to TLR3 agonist poly(I:C), to mimic the endogenous TLR3 agonist stathmin present in myelin debris^49^ or a mix of pro-inflammatory cytokines IFN-γ, TNF-α, and IL-1β to recapitulate the inflammatory environment in GMLs.^50^^,^^51^ In untreated conditions, TNC expression was not different between PPMS-iAstro and PPsib-iAstro (Figures 4A and 4B). Although not significant, TNC expression tended to increase upon treatment with either poly(I:C) or pro-inflammatory cytokines, which was more pronounced for PPsib-iAstro (Figures 4A–4C and 4D). Hence, although not statistically significant as of the low sample size, a pro-inflammatory cytokine environment appeared to increase rather than decrease TNC expression, with a slightly lower response in PPMS-iAstro compared to PPsib-iAstro. Although iAstro cannot fully recapitulate the complex cellular environment in GMLs, these findings suggest that the lower abundance of TNC in dGMLs with a high number of HLA-DR+ microglia is unlikely to be explained by decreased astrocytic TNC expression under inflammatory conditions.

### Altered microglia characteristics on decellularized subpial GMLs

**Figure 5:**
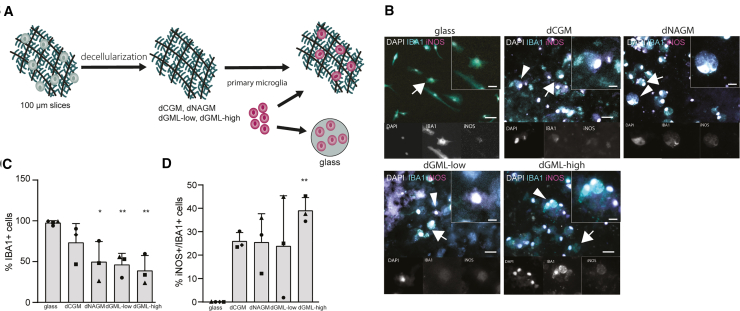
Altered microglia characteristics on decellularized subpial GMLs (A) Experimental set-up. Primary rat microglia were cultured on uncoated glass or introduced to 100 μm slices of decellularized control gray matter (dCGM), normal appearing gray matter (dNAGM), and subpial gray matter lesions with low (dGML-low) or high (dGML-high) HLA-DR+ microglia numbers. (B) Representative images of microglia cultured on the indicated substrates. Microglia are co-stained for microglia marker IBA1 (cyan) and pro-inflammatory marker iNOS (magenta). Nuclei are visualized with DAPI (white). Scale bars are 10 μm (overview) and 5 μm (inset). Arrow indicates iNOS- IBA1+ cells, arrowheads depict iNOS+ IBA1+cells. (C and D) Quantification of the percentage of IBA1+ cells of total cells (DAPI, C) and percentage of iNOS+ cells of total IBA1+ cells (D). Bars represent mean values. Error bars represent standard error of the mean. Dots represent independent cell culture experiments on tissues of different donors. Statistical analysis was performed using one-way ANOVA with Tukey’s multiple comparisons test to test for differences between groups (C,D, ∗*p* < 0.05, ∗∗*p* < 0.01).

As the ECM, including TNC, is involved in microglia homeostasis and response to injury,^39^^,^^52^^,^^53^^,^^54^ we next examined the effect of the non-cell-associated ECM on microglia, by repopulating decellularized CGM, NAGM, and GML tissue slices with primary microglia (Figure 5A). When introduced to decellularized GM tissue, microglia adhered and were evenly distributed over the slices. However, where a predominantly ramified morphology was observed when cultured on glass, microglia seem to adopt an amoeboid morphology on decellularized GM tissue (Figures 5B and S5). Surprisingly, the percentage of cells positive for microglia marker IBA1 was significantly lower when microglia were cultured on dNAGM, dGML-low, and dGML-high but not dCGM compared to uncoated glass (2-fold decrease, Figure 5C). Notably, on glass approximately 98% of the cells were IBA1+. Furthermore, while inducible nitric oxide synthase (iNOS)+/IBA1+ cells were not detected when cultured on glass, on decellularized GM tissue, 30%–40% of the IBA1+ cells also expressed pro-inflammatory marker iNOS^55^^,^^56^ (Figure 5D). The percentage of iNOS+/IBA1+ cells was significantly higher when microglia were cultured on dGML-high compared to microglia grown on uncoated glass (Figure 5D). Notably, iNOS+/IBA1- cells were not observed. These findings indicate that the non-cell-associated ECM of MS gray matter tissue, even before notable demyelination, impacts microglia characteristics by reducing the percentage of IBA1+ cells, while a subset of the remaining IBA1+ microglia acquired iNOS expression.

## Discussion

In this study, we examined the largely unexplored ECM in gray matter affected by MS, a critical component for understanding disease progression, using decellularized tissue slices, discovery proteomics, and microglia repopulation assays. Our findings indicate prominent alterations in the non-cell-associated ECM composition in non-demyelinated cortex of people with MS, while the non-cell-associated ECM of GMLs alter microglia characteristics. These findings add to our insights in gray matter MS pathology, including neuroinflammation and synaptopathy.

Proteomic profiling revealed differential abundances of four ECM components of dNAGM compared to dCGM. Specifically, NCAN and HPLN1 were more abundant; VCAN and TNR were less abundant in dNAGM. Discovery proteomics indicated that NCAN and HPLN1 were less abundant in the cuprizone-demyelinated decellularized cortex, which remains to be validated by western blotting. Assuming that the molecular response to cuprizone-induced demyelination reflects that of demyelination in humans, the enriched abundance of NCAN and HPLN1 in dNAGM does likely not represent early demyelination but rather constitutes a distinct feature of non-demyelinated MS gray matter tissue. However, as NCAN and HPLN1 may be transiently more abundant at early time points than 3 weeks cuprizone feeding, we cannot exclude that NCAN and HPLN1 are more abundant in dNAGM as a compensatory mechanism at the onset of demyelination.

The four differential differentially abundant proteins are key PNN components. The CSPGs NCAN and VCAN are linked via HPLN1 to HA, which serves as a critical scaffold for PNNs, while TNR further stabilizes the network by crosslinking CSPGs.^57^ Although the spatial localisation of the alterations in ECM composition of decellularized tissue remains to be determined and cellular components are not completely eliminated, it is tempting to suggest that the contrasting abundances of PNN components observed in dNAGM and dCGM indicate a perturbed PNN composition rather than PNN loss and likely an altered interaction between ECM constituents. In agreement with this line of reasoning, Gray et al., reported no evident loss of PNNs surrounding parvalbumin-expressing neurons in non-demyelinated MS cortex.^58^ Furthermore, changes in PNN-component composition likely leads to changes in PNN structure and function, including synaptic plasticity and stabilization. Western blot analysis confirmed a significant increase in HPLN1 and a decrease in VCAN levels in dNAGM compared to dCGM. VCAN promotes presynaptic maturation,^59^ facilitates leukocyte infiltration,^60^^,^^61^ and interacts with macrophages, either directly or indirectly via HA, to enhance pro-inflammatory cytokine secretion.^62^^,^^63^^,^^64^ Therefore, downregulation of VCAN levels may represent a compensatory mechanism aimed at protecting neuronal loss and preventing demyelination. In addition, the presence of both LGI1 and its receptor ADAM22 in dNAGM, but not dCGM, suggests excitatory synapse maturation, as LGI1 regulates the functional incorporation of postsynaptic PSD95 via ADAM22.^65^ The exclusive detection of PLXNA4 in all dNAGM further supports synapse maturation. PLXNA4 is involved in excitatory postsynaptic signaling as a receptor for endothelial-derived semaphorin 3G in hippocampal neurons.^66^ Finally, the presence of matrisome proteins TINAGL1 and HSPG2 in the dNAGM, but not dCGM proteome, both associated with the blood-brain barrier (BBB) endothelium, points to potential changes in basement membrane composition. Hence, these findings suggest that the pathological alterations in non-cell-associated ECM composition in absence of demyelination may reflect broad changes in PNNs and synapse- and BBB-associated ECM within NAGM. However, the construction of proteome maps at the single cell level using spatial quantitative proteomics is required to allow for a direct assessment of local alterations in ECM components at PNNs, around synapses, at the BBB, or in the interstitial matrix. From a diagnostic and translational perspective, if alterations in the non-cell-associated ECM map to PNNs and BBB, such signatures could potentially serve as biomarkers of gray matter pathology.

While PNNs are lost in the demyelinated MS cortex as a consequence of MMP9 activity,^58^ our quantitative proteomic analysis revealed comparable levels of NCAN and HPLN1 in decellularized subpial GMLs and surrounding non-demyelinated PLGM. TNC, highly present in neurogenic niches and injured areas^67^^,^^68^^,^^69^^,^^70^ was less abundant in dGMLs with high microglia activity. TNC is an endogenous activator of TLR4, regulating chemotaxis, phagocytosis, and proinflammatory cytokine production in microglia.^52^ In the ischemic brain, lack of TNC led to reduced microglia surveillance in perilesional non-injured areas, favoring leukocyte accumulation.^53^ Notably, FGB and BGN, which are uniquely detected in dGMLs, are also endogenous TLR4 agonists,^71^^,^^72^^,^^73^ and an indication of BBB integrity loss.^74^ Why TNC abundancy was lower in dGMLs with high HLA-DR+ microglia numbers in native tissue remains to be determined. TNC is primarily produced by astrocytes, and although TNC expression was potentially less increased in PPMS-iAstro compared to non-affected PPsib-iAstro upon exposure to TLR3 agonist poly(I:C) or a mix of pro-inflammatory cytokines, the protein levels were not reduced. Potentially, the lower abundancy of TNC in dGMLs with high HLA-DR+ microglia presence may result from HLA-DR+ microglia that secrete TNC-degrading MMPs,^75^ although the expression and activity of MMPs in GMLs remains to be explored.

Reciprocally, the ECM influences microglia as microglia respond to both mechanical signals, such as stiffness, and biological signals from the ECM.^39^^,^^54^^,^^76^^,^^77^^,^^78^ When introduced to decellularized tissue slices, microglia seem to adopt an ameboid morphology on control and MS GM tissue slices but appeared more ramified morphology on uncoated glass. These apparent morphological changes likely relate to differences in stiffness between glass and decellularized tissue slices.^77^^,^^78^^,^^79^ Glass has a Young’s modulus of approximately 50–90 GPa,^80^ whereas native CGM and NAGM tissue slices measure about 0.5 kPa. Stiffness measurements for native GMLs and decellularized gray matter tissue were not feasible due to probe entrapment in the soft tissue. On decellularized MS gray matter tissue, but not on decellularized CGM, the percentage of IBA1+ microglia decreased compared to glass, while the proportion of iNOS+ cells among the remaining IBA1+ cells significantly increased for microglia plated on dGMLs with high HLA-DR+ microglia numbers in native tissue. Notably, a previous study revealed that CD206, but not iNOS, mRNA levels increased in microglia plated on a soft substrate.^78^ This suggests that the increase in iNOS+ IBA1+ microglia likely does not relate to stiffness, but reflects pathological differences in decellularized GML composition. Hence, our data highlight that microglia are affected by the deposited ECM in subpial GMLs, potentially mimicking a pro-inflammatory state. However, although decellularized tissue slices preserve the native non-cell-associated ECM topological architecture, their limited availability and fragile nature did not allow for thorough analysis of functional microglia properties, such as proliferation, phagocytosis, and ECM-remodelling. Therefore, further investigation into the complex interplay between ECM stiffness and composition and their impact on MS microglia function and behavior is required, for example using MS-iPSC-derived microglia cultured on hydrogels with tuneable mechanical properties incorporating brain ECM or brain-derived ECM hydrogels,^81^^,^^82^ to better understand the relevance to MS pathology.

Taken together, our findings highlight significant alterations in the ECM composition of decellularized MS GM particularly in the absence of detectable demyelination, potentially influencing microglia and contributing to pathology. Moreover, altered abundances in PNN ECM components in dNAGM and uniquely detected BBB-associated components in dNAGM and dGMLs suggest a broader impact on synaptic function and BBB integrity. Further research is needed to understand how abnormalities in non-cell-associated ECM and reciprocal ECM-microglia interactions contribute to neuroinflammation, synaptopathy, and neuronal dysfunction in MS.

### Limitations of the study

As outlined in the discussion, this study has a few limitations. First, due to the study design, (decellularized) CGM vs. NAGM and PLGM vs. GML tissue slices were obtained at different time points and analyzed in separate LC-mass spectrometry runs. This introduces a potential batch effect, and as decellularized tissue does not permit reliable protein coverage comparisons, quantitative analyses were restricted to samples that were processed in the same batch. Therefore, direct quantitative comparisons between dNAGM and dGMLs were not feasible and will require future experiments using new samples from additional donors. Second, the observed alterations in ECM composition have not yet been spatially validated to PNNs, BBB, synapses, and/or interstial matrix. Addressing this will require spatial proteomic approaches with subcellular resolution. Third, although our optimized decellularization protocol substantially reduced the DNA content to levels comparable to reported for other organs, complete removal of cellular components was not achieved, which may have affected microglia characteristics. More aggressive physical methods were not feasible due to the inherent structural fragility of brain tissue, and extended washing or prolonged detergent exposure led to tissue disintegration. Notably, damage-associated molecule patterns (DAMPs) potential residing inside the decellularized tissue, such as nuclear protein HMGB1, were not detected in any of our proteomic analyses. Fourth, the evaluation of the effect of decellularized tissue on microglia was limited to morphology and immunostainings, and therefore does not provide direct insight into functional changes in microglia. Future experiments using hiPSC-derived microglia rather than primary microglia are required to disentangle the relative contributions of mechanical cues, biological signals, or their combination in mediating the effect of decellularized tissue on microglia characteristics. In addition, direct functional assays of microglia, such as cytokine secretion and phagocytic capacity, are needed to determine how non-cell-associated ECM contributes to gray matter pathology in MS.

## Resource availability

### Lead contact

Requests for further information and resources should be direct to and will be fulfilled by the lead contact, Wia Baron (w.baron@umcg.nl).

### Materials availability

This study did not generate new unique reagents.

### Data and code availability

• Proteomic datasets generated and analyzed in the current study are available through the Proteomics IDEntification Database (PRIDE) repository under accession number PXD066920.
• This study does not report original code.
• Any additional information required to reanalyse the data reported in this study is available from the lead contact upon request.

## Acknowledgments

We would like to thank Naomi Dijksman and Dr. Hilmar van Weering for their excellent technical assistance and Dr. Patrick van Rijn for the useful discussions on stiffness measurements of brain tissue slices. This work was supported by the Dutch MS Research Foundation (Stichting MS Research, 18-733c MS and 22-733d, MSCNN program grant and 18-1001 MS, out-of-the-box grant made possible by MoveS, B.J.L.E., and W.B.) and 10.13039/100016060Stichting de Cock-Hadders (2021-52, J.M.d.J.).

## Author contributions

J.M.d.J., investigation, formal analysis, project administration, visualisation, and writing – original draft; J.C.W., methodology and writing – reviewing and editing; M.H.C.W., investigation, writing – reviewing and editing; J.d.V., investigation and writing – reviewing and editing; S.M.K., supervision and writing – reviewing and editing; B.J.L.E., supervision, funding acquisition, and writing – reviewing and editing; W.B., conceptualization, project administration, supervision, funding acquisition, and writing – reviewing and editing; all authors have read and approved the manuscript.

## Declaration of interests

J.M.d.J. is currently employed as a contractor by Elsevier as associate scientific editor at Data in Brief and MethodsX. The author contribution to this study was performed prior to joining Elsevier during her PhD training at the University of Groningen. J.M.d.J had no involvement in the editorial handling or peer-review process of this manuscript.

## STAR★Methods

### Key resources table

**Table:** 

REAGENT or RESOURCE	SOURCE	IDENTIFIER
**Antibodies**		

anti-β-actin	Sigma-Aldrich	Cat# A5441 RRID:AB_476744
anti-H3	Abcam	Cat# ab1791 RRID:AB_302613
anti-HLA-DR	Thermo Fisher Scientific	Cat# 14995682 RRID:AB_468639
anti-IBA1	Wako	Cat# 019-19741 RRID:AB_839504
anti-iNOS	BD Bioscience	Cat# 610329 RRID:AB_397719
anti-laminin 1 + 2	Abcam	Cat# ab7463 RRID:AB_305933
anti-PLP	Bio-Rad	Cat# MCA839G RRID:AB_2237198
anti-TNC	Abcam	Cat# ab108930 RRID:AB_10865908

**Biological samples**		

Human control and multiple sclerosis brain tissue	Netherlands Brain Bank	https://www.brainbank.nl/
Primary microglia	RccHan:WIST, Envigo	RRID:RGD_5508396

**Chemicals, peptides, and recombinant proteins**		

cuprizone	Sigma-Aldrich	Cat# C9012
CHAPS hydrate	Sigma-Aldrich	Cat# C3023
Triton X-100	Arcos	Cat# CAS 9002-93-1
Sodium deoxycholate	Sigma-Aldrich	Cat# D6750
benzonase	Merck	Cat# E1014
DNase	Roche	Cat# 10104159001
poly(I:C)	Sigma-Aldrich	Cat# P1530
IFNγ	Peprotechs	Cat# 300-02
TNFα	Peprotech	Cat# 300-01
IL1β	Peprotech	Cat# 200-01

**Critical commercial assays**		

sulfated glycosaminoglycan (sGAG) assay	Chondrex	Cat# 6022
HA Quantikine ELISA kit	R&D systems	Cat#DHYAL0
Hydroxyproline assay kit	Chondrex	Cat# 6017
DC Bio-Rad protein assay kit	Bio-Rad	Cat# 500-0114

**Deposited data**		

Raw and analyzed data	this paper	Tables 1, S2, S3, and S5 PRIDE: PXD066920

**Experimental models: Cell lines**		

hiPSC	MSiPS Biobank	https://www.msips-biobank.nl

**Experimental models: Organisms/strains**		

C57BL/6J	Envigo	N/A

**Software and algorithms**		

Image Studio Lite	Image Studio software	https://www.licorbio.com/
GraphPad Prism	GraphPad Software	https://www.graphpad.com
Leica Application Suite Advanced	Leica Application Suite Advanced Fluorescence software	https://www.leica-microsystems.com/
MaxQuant	MaxQuant software	https://maxquant.org/
Perseus	MaxQuant software	https://maxquant.net/perseus/
Gene Ontology	Gene Ontology	https://www.geneontology.org/

**Other**		

Millicell cell culture inserts	Merck	Cat# PICM3050

### Experimental model and study participant details

#### Animal studies

Mice (C57BL/6J, Envigo, the Netherlands) and (pregnant) rats (RccHan:WIST, Envigo, the Netherlands) were individually housed on a 12/12h light/dark cycle (7.00/19.00 lights on/off) with *ad libitum* access to food and water. Animal protocols were approved by the central committee on animal experiments (CCD, rats: AVD10500202216102; mice: AVD105002016504) in the Netherlands and the institutional animal ethics and care committees (DEC, IvD, 2216102-01-001 and 16504-04-02) of the University of Groningen, and carried out in accordance with the European Council Directive (2010/63/EU) on the protection of animals used for scientific purposes.

#### Primary microglia

Mixed glial cells were isolated from cortical and non-cortical areas of pooled female and male neonatal (postnatal day 1–3) RccHan:WIST rat brains as described.^83^ Briefly, mixed glial cells were cultured for 13 days in DMEM (#41965, Gibco) supplemented with 10% non-heat inactivated fetal bovine serum (FBS, #FBS-12A, Capricorn Scientific), 100 U/ml penicillin and 100 μg/mL streptomycin (#15140, Invitrogen), and 4 mM L-glutamine (#25030, Invitrogen). To enrich for microglia, a shake-off procedure was used.^83^ Briefly, flasks containing mixed glial cultures were shaken in an orbital shaker (Innova 4000, New Brunswick) at 150 rpm for 1 h at 37°C. Medium was collected and centrifuged for 5 min at 1300 rpm. Microglia cultured in microglia medium [100 U/ml penicillin and 100 μg/mL streptomycin, 4 mM L-glutamine, 1 mM pyruvate (#11360-039, Gibco), 10% (v/v) non-heat inactivated FBS in DMEM] for 2 days on 13-mm glass coverslips in a 24 well plate at a density of 40,000 cells per well or on decellularized GM tissues in Millicell cell culture inserts (30 mm, #PICM03050, Merck) in a 6-well plate at a density of 500,000 cells per insert.

#### Cell lines

Human induced pluripotent stem cells from people with primary progressive MS (PPMS1, MSB036-CL01, female; PPMS2, MSB019-CL02, male; PPMS3, MSB012-CL05, male) and their non-affected siblings (PPsib1, MSB035-CL01, female; PPsib2, MSB018-CL02, female, PPsib3, MSB014-CL01, male) were obtained from the MSiPS biobank (UMCG, the Netherlands, https://www.msips-biobank.nl). hiPSCs were generated using non-integrative reprogramming of peripheral mononuclear blood cells, have normal karyotypes, are pluripotent and free of mycoplasma. hiPSCs were cultured on matrigel (#354277, Corning) in mTeSR Plus (#100–0276, Stemcell technologies). hIPSCs were passaged when the colonies reach a size of 100–250 μm and detached using ReLeSR (#05872, Stemcell technologies).

### Method details

#### Snap-frozen postmortem human brain tissue

Snap-frozen frozen postmortem human brain tissues of MS and non-demented control donors were obtained from the Netherlands Brain Bank (NBB). CGM and NAGM were selected based on the absence of demyelinating and inflammatory pathology, as assessed by immunohistochemical analysis of PLP (myelin) and HLA-DR (microglia/macrophages), respectively. GMLs were included based on subpial demyelination comprising only the upper cortical layers or spanning the entire width of the cortex without extending in the subcortical WM.^84^ Subpial GMLs were scored for HLA-DR+ cells, which are mainly microglia,^85^ and divided in GML-low when only few HLA-DR+ cells were present in the demyelinated area and GML-high when many HLA-DR+ cells were detected. In the surrounding non-demyelinated PLGM only few HLA-DR+ cells were present. The age, female/male ratio and postmortem delay were comparable between non-demented control and MS donors (Table 1). Informed consent was given by the donors for brain autopsy and the use of tissue for the purpose of scientific research and approved by the Ethical Committee of the VU University Medical Center Amsterdam (2019/148).

#### Cuprizone-induced demyelination

To induce demyelination, 16 8-week-old male C57BL/6 mice were fed with 0.2% (w/w) cuprizone (#C9012, Sigma-Aldrich) mixed with water in powdered normal chow.^86^^,^^87^ The cortex was collected after 3 weeks cuprizone (demyelinat*ing*, *n* = 4), 5 weeks cuprizone (demyelinat*ed*, *n* = 4) feeding, and 5 weeks cuprizone feeding followed by 1 week of normal chow (remyelina*ting*, *n* = 4) or 2 weeks of normal chow (remyelinat*ed*, *n* = 4). 4 8-week-old male C57BL/6 mice were fed with normal chow (control, *n* = 4). Mice were randomly assigned to experimental groups. Brains were collected, the cortex was dissected from the rest of the brain, frozen in liquid nitrogen and stored at −80°C. Notably, this study is limited by the use of only male animals.

#### Atomic force microscopy

CGM and NAGM tissue were snap-frozen in liquid nitrogen and sectioned in 100 μm slices using a cryostat and glued to a microscopy slide with transparent nail polish. Unfixed slices were kept in PBS and measurements were performed within 3 h. Stiffness measurements were performed in PBS with an AFM (Bruker Catalyst) using a cylindrical tip with a radius of 10 μm (SAA-SPH0-10UM, Bruker) with a pre-calibrated spring constant around 0.2 N/m. Samples were measured in three distinct regions spread out over the target area, on 30 force curves per region with 10 μm in between the spots. Force curves were recorded with a loading speed of 10 μm/s, and a loading force of 5 nN. From the force curves the Young’s modules was calculated by dividing the applied stress by the physical deformation (or strain), resulting in Young’s modulus in kPa (1 kPa = 1 N/m^2^) with the software package NanoScope Analysis (version 1.80) (Brüker). For the modulus calculations the Hertz model (spherical indenter) was used. To ensure consistency across samples, all measurements were performed at the same day, using the same cantilever and identical acquisition parameters. Of note, we cannot exclude that snap-freezing in liquid nitrogen may influence absolute mechanical properties, but as all tissues were identically processed relative comparisons can be performed.

#### Decellularization

##### Human gray matter brain tissue

Snap-frozen postmortem human gray matter tissue was sectioned in 100 μm slices using a cryostat. For GML-containing slices, lesions were manual dissected from perilesional areas. Dependent on the size of the tissue, one or two slices were placed on Millicell cell culture inserts. To confirm similar classification before and after sectioning, consecutive 12 μm sections were stained for PLP (myelin) and HLA-DR. Tissue slices were decellularized according to our optimized protocol for thin gray matter human brain slices.^44^ To solubilize cell membranes and dissociate DNA from proteins, slices were treated with 4 mM 3-((3-cholamidopropyl) dimethylammonio)-1-propanesulfonat (CHAPS hydrate, #C3023, Sigma-Aldrich) in dH_2_O overnight at room temperature (RT). Next, to efficiently disrupt lipid-lipid and lipid-protein interactions, slices were subsequently incubated with 3% Triton X-100 (#CAS 9002-93-1, Arcos) for 1 h at RT and washed twice with dH_2_O. To remove nucleotides after cell lysis, slices were incubated with 0.02 U/ml benzonase (#E1014, Merck) in phosphate buffered saline (PBS) supplemented with 1 mM MgCl_2_ for 1 h at RT. To further disrupt cell membranes and denature proteins, slices were treated with 2% sodium deoxycholate (DOC, #D6750, Sigma-Aldrich) for 1 h at RT and subsequently washed twice with dH_2_O and twice with PBS. The decellularized slices were used the same day or stored on inserts in PBS at 4°C for a maximum of two days.

##### Mouse cortex tissue

Snap-frozen cortex was sectioned in 100 μm slices using a cryostat (Leica CM3050S) and slices were placed on Millicell cell culture inserts (four cortices/insert). Slices were treated with 4 mM CHAPS hydrate in dH_2_O overnight at RT, subsequently washed twice with dH_2_O, and incubated with 40 μg/mL DNase (#10104159001, Roche) in PBS for 1 h at 37°C to remove DNA after cell lysis. Slices were treated with 2% DOC in dH_2_O for 1 h at RT and washed twice with dH_2_O and twice with PBS. The decellularized slices were used the same day or stored on the cell culture inserts in PBS at 4°C for a maximum of two days.

##### Processing

Decellularized slices were transferred into an Eppendorf tube and lysed with TNE buffer containing 10 mM Tris base (#T6066, Sigma-Aldrich), 100 mM NaCl, 1 mM EDTA, 1% Triton X-100 (#9002-93-1, Acros) and a complete mini protease inhibitor cocktail per 10 mL buffer (#11836153001, Roche Diagnostics GmbH). The content of the Eppendorf was equally divided, half was used for mass spectrometry analysis and the other half for validation studies. For the microglia repopulation experiments on the human tissues, the decellularized slices were incubated with microglia culture medium and kept in 37°C for two to four days prior to cell seeding.

#### Protein and DNA content

Native and decellularized tissue slices were homogenized in 500 μL TNE buffer using a handheld tissue homogenizer.

##### Protein content

To determine the protein content of the native and decellularized homogenates a DC Bio-Rad protein assay (#500-0114, Bio-Rad) was used with BSA as standard according to manufacturer’s instructions. Absorbance was measured at 750 nm (BioTek Synergy HTX plate reader).

##### DNA content

Tissue homogenates were mixed 1:20 (v/v) with a buffer containing 100 mM EDTA, 50 mM Tris, 1% SDS, 100 mM NaCl, 0.2% proteinase K (#19131, Qiagen) and dH_2_O and incubated overnight at 55°C. NaCl was added to a final concentration of 1.43 M and the samples were centrifuged at 15,500 g for 10 min at RT. The resulting supernatant was diluted 2:1 (v/v) with isopropanol (#1.09634.1000, Sigma-Aldrich), and centrifuged at 15,500 g for 10 min at RT. The pellet was washed with 70% ethanol, centrifuged at 15,500 g for 10 min at RT, airdried and resuspended in 20 μL RNase-free dH_2_O. DNA concentration was measured using a Nanodrop 2000/2000c spectrophotometer (Thermo Scientific). The DNA content is expressed as ng/μg protein of the native slice.

#### Proteomics

##### Mass spectrometry sample preparation

Protein levels of decellularized tissue slice homogenates were determined using discovery-based proteomics (using label free quantification) on equal protein concentrations.^88^ Briefly, the pellet was resuspended in 50 μL NP40 buffer (0.1% Nonidet P-40 (NP-40), 0.4 M NaCl, 10 mM Tris-HCl (pH 8.0), 1 mM EDTA) and lysed using bead beating (Precellys 24 bead beater: program 6000 rpm, three times 15 s, 15 s break) or bath sonication (15 min). In-gel digestion was performed on the lysed samples using trypsin (1:100 g/g sequencing grade modified trypsin (#V5111; Promega) after reduction with 10 mmol/L dithiothreitol and alkylation with 55 mmol/L iodoacetamide proteins as described previously.^89^

##### Discovery-based mass spectrometric analyses

Discovery mass spectrometric analyses were performed on a quadrupole orbitrap mass spectrometer equipped with a nano-electrospray ion source (Orbitrap Q Exactive Plus, Thermo Scientific). Chromatographic separation of the peptides was performed by liquid chromatography (LC) on a nano-HPLC system (Ultimate 3000, Dionex) using a nano-LC column (Acclaim PepMapC100 C18, 75 μm × 50 cm, 2 μm, 100 Å, Dionex, buffer A: 0.1% v/v formic acid, dissolved in milliQ-H_2_O, buffer B: 0.1% v/v formic acid, dissolved in acetonitrile). In general, half of the digest was injected using the μl-pickup method with buffer A as a transport liquid from a cooled autosampler (5°C) and loaded onto a trap column (μPrecolumn cartridge, Acclaim PepMap100 C18, 5 μm, 100 Å, 300 μmx5 mm, Dionex). Peptides were separated on the nano-LC column using a linear gradient from 2 to 45% buffer B in 86 min at a flowrate of 300 nL/min. The mass spectrometer was operated in positive ion mode and data-dependent acquisition mode (DDA) using a top-15 method. MS spectra were acquired at a resolution of 70,000 at m/z 200 over a scan range of 300–1650 m/z with a AGC target of 3e^6^ ions and a maximum injection time of 50 ms. Peptide fragmentation was performed with higher energy collision dissociation (HCD) using a normalized collision energy (NCE) of 28. The intensity threshold for ions selection was set at 2.0 e^4^ with a charge exclusion of 1≤ and ≥6. The MS/MS spectra were acquired at a resolution of 17,500 at m/z 200, a AGC target of 1e^3^ ions and a maximum injection time of 50 ms and the isolation window set to 1.8 m/z.

LC-MS raw data were processed with MaxQuant (version 1.5.5.1).^90^ Peptide and protein identification were carried out with Andromeda against a human SwissProt database (www.uniprot.org, canonical database 20350 entries). Proteins were quantified with the MaxLFQ algorithm,^91^ including both razor and unique peptides and a minimum ratio count of two. The results were imported in Perseus (version 1.5.5.3)^92^ for further processing and filtering. Protein groups were filtered for proteins that were quantified in at least two biological replicates in both groups. Gene ontology (GO) over-representation analysis for biological process was performed on the full protein datasets without differential comparison using a GO term analysis tool and the PANTHER knowledgebase.^93^^,^^94^ A computationally assembled ECM proteome, termed the matrisome, consisting of ECM-core and ECM-associated proteins was used to identify ECM components.^47^ Matrisome proteins were considered to be common if they were detected in at least two out of four samples across tissue groups. If matrisome proteins were detected in at least two out of four samples but only within a single tissue group, they were assigned as uniquely present matrisome proteins. Notably, CGM and NAGM, and PLGM and GMLs were analyzed in separate LC-mass spectrometry runs, and can therefore not be compared.

#### ECM quantification assays

##### Sulfated glycosaminoglycans

To quantify the sulfated glycosaminoglycan (sGAG) content in native and decellularized human tissue slices, a sGAG assay kit *(#*6022, Chondrex) was used according to manufacturer’s instructions. The kit utilizes an enhanced solution of 1,9 dimethylmethyelene blue, a cationic dye that specifically binds to highly sGAGs but not to hyaluronan (HA), while its interaction with negatively charged DNA and RNA is minimal. Absorbance was measured at 520 nm (BioTek Synergy HTX plate reader). The sGAG content is expressed as ng/μg total protein.

##### Hyaluronan

An HA Quantikine ELISA kit (#DHYAL0, R&D systems) was used to quantify the amount ofHA in native and decellularized human tissue slices, according to manufacturer’s instructions. HA greater than or equal to 35 kDa that binds to immobilized recombinant human aggrecan is measured by this quantitative sandwich enzyme immunoassay. Absorbance was measured at 450 nm (BioTek Synergy HTX plate reader). The HA content is expressed as ng/μg total protein.

##### Collagen

To quantify the amount of collagen in native and decellularized human tissue slices a hydroxyproline assay kit (#6017, Chondrex) was used according to manufacturer instructions. This kit detects all collagen types. Absorbance was measured at 540 nm (BioTek Synergy HTX plate reader). The collagen content is expressed as ng/μg total protein.

#### hiPSC-derived astrocytes

hiPSCs were differentiated into neural progenitor cells (iNPCs) by culturing the cells in neural induction medium (DMEM-F12, MEM-NEAA (1:100, #11140-035, Thermofisher Scientific), 2 mM L-glutamine (#25030-024, Thermofisher Scientific), penicillin-streptomycin (#100 U/ml, Thermofisher Scientific), 2-mercaptoethanol (1 μg/mL, #M3148, Sigma-Aldrich), insulin (100 μg/mL, #12585014, Gibco)), supplemented with 100 nM retinoic acid (#R2625, Sigma-Aldrich), 10 μm SB-431542 (#1614, R&D Systems) and 250 nM LDN-193189 (#04–0074, Stemgent). Upon culturing in astrocyte medium iNPCs were differentiated into astrocytes (iAstro) (#1801, ScienceCell Research Laboratories) in 30 days, as described.^95^ iAstro were plated on 6-well plates (200,000 cells/well). Cells were left or treated with TLR3 agonist poly(I:C) (50 μg/mL, #P1530, Sigma-Aldrich) or a pro-inflammatory cytokine mix containing IFNγ (1000 U/ml, #300-02, Peprotech), TNFα (20 ng/mL, #300-01A, Peprotech) and IL1β (20 ng/mL, #200-01B, Peprotech) for 48 h.

#### Western blot

Equal protein amounts (20 μg) from the native slices, tissue homogenates, iAstro lysates or decellularized slices (for ECM analysis) or an equivalent volume from the decellularized slices (for the validation of decellularization efficiency) were mixed with 4X SDS-reducing sample buffer to reach a final concentration of 1X. Samples were heated at 95°C for 5 min. The samples were loaded onto a 10% (NCAN, VCAN, HPLN1, H3, TNR, actin) or 8% (TNC, laminin, actin) SDS-polyacrylamide gel and ran at 120V. Proteins were transferred onto a PVDF membrane (Immobilon-FL PVDF, 0.45 μm, #IPFL85R, Millipore) using a wet transfer system (Bio-Rad) for 1 h at 500 mA (NCAN, VCAN, HPLN1, H3, TNR, actin) or a semi-dry transfer system (Bio-Rad) for 40 min at 15V (TNC, laminin, actin). The membranes were blocked with blocking buffer (#927–70001, LI-COR) diluted 1:1 in PBS for 1 h on a shaker at RT and subsequently incubated with primary antibodies (Table 2) in blocking buffer (1:1 with PBS) supplemented with 0.1% Tween 20 (PBS-T) overnight at 4°C. The membranes were washed three times in PBS-T, and incubated with the appropriate secondary antibodies (Table 2) in blocking buffer (1:1 in PBS-T) for 1 h at RT. After three washes with PBS-T, the membranes were scanned with the Odyssey CLx Imaging System and the intensity of the visualized bands quantified using LI-COR Image Studio Lite software (version 5.2). To allow quantitative comparisons between samples on different blots, a reference sample was taken along on all gels and set to 1. Raw images of the immunoblots are provided in Figure S6.

**Table:** Primary and secondary antibodies used for ICC, IHC, and WB

protein^a^	host	dilution ICC	dilution IHC	dilution WB	catalog #	manufacturer
β-actin	ms	1:1000	n.a.	1:1000	A5441	Sigma-Aldrich
H3	rb	n.a.	n.a.	1:1000	ab1791	Abcam
HLA-DR	ms	n.a.	1:500	n.a.	14995682	Thermo Fisher Scientific
IBA1	rb	1:500	n.a.	n.a.	019–19741	Wako
iNOS	ms	1:250	n.a.	n.a.	610329	BD Bioscience
laminin 1 + 2	rb	1:1000	n.a.	1:1000	ab7463	Abcam
PLP	ms	n.a.	1:400	n.a.	MCA839G	Bio-Rad
TNC	rb	n.a.	n.a.	1:250	ab108930	Abcam
α-rb 680	gt	n.a.	n.a.	1:10,000	925–68071	LI-COR
α-ms 800	gt	n.a.	n.a.	1:10,000	925–32210	LI-COR
α-rb 594	dk	1:500	n.a.	n.a.	A21207	Invitrogen
α-ms 488	dk	1:500	n.a.	n.a.	A21202	Invitrogen
α-rb 488	gt	1:500	n.a.	n.a.	A11034	Invitrogen
α-ms 594	gt	1:500	n.a.	n.a.	A21263	Invitrogen
α-rb TRITC	gt	1:50	n.a.	n.a.	111-025-003	Jackson ImmunoResearch
biotin-conjugated α-ms	hs	1:400	n.a.	n.a.	BA-2001	Vector Laboratories
biotin-conjugated α-rb	gt	1:400	n.a.	n.a.	BA-1000	Vector Laboratories

a ms = mouse, rb = rabbit, gt = goat, dk = donkey, hs = horse, α = anti, n.a. = not applicable.

#### Immunochemistry

##### Immunohistochemistry

Immunohistochemical staining for PLP and HLA-DR were performed on 12 μm slices of snap-frozen tissue cut on a cryostat and dehydrated for 30 min in an exicator. Sections were rinsed with PBS and incubated with 0.3% H_2_O_2_ to block endogenous peroxidase. After washing three times with PBS, sections were incubated with primary antibodies (Table 2) in PBS with 2% normal horse serum overnight at 4°C. The next day, sections were washed and incubated with the appropriate secondary antibodies (Table 2) for 2 h at RT. The sections were washed with PBS and incubated with ABC solution (#PK-4000, Vector Laboratories) for 20 min. After washing three times with PBS, sections were incubated with 3,3′-diaminobenzidine (DAB, 1:50 in PBS, #D5637, Merck) and 1.5% H_2_O_2_ for 10 min on RT. Sections were counterstained with Mayer’s Haematoxylin (#MHS16, Merck) for 30 s, dipped in tap water and dehydrated in an ethanol series, air dried and mounted with DePeX (#18243, Serva).

##### Immunocytochemistry

Cells were fixed with 4% paraformaldehyde (PFA) in PBS for 20 min at RT. Non-specific antibody binding was blocked with 4% bovine serum albumin (BSA, #A7030, Sigma-Aldrich) in PBS for 30 min. Cells were incubated with primary antibodies (Table 2) overnight in PBS with 2% BSA at 4°C. Cells were washed three times with PBS and incubated for 1 h with appropriate secondary antibodies (Table 2) and DAPI (2 μg/mL, #32670, Sigma-Aldrich) in PBS. Omission of the primary antibody showed no specific signal. After washing three times with PBS, cells were mounted with mounting medium (#S3025, Dako). Cells were visualized and manually counted in a blinded manner using a Leica DM6000b fluorescent microscope equipped with Leica Application Suite Advanced Fluorescence software. Raw images of the microscopy images are provided in Figures S7 and S8. A minimum of 300 cells was counted per coverslip or tissue (area). The percentage of IBA1+ cells was calculated out of total cells (DAPI), and the percentage of proinflammatory marker iNOS+ cells out was calculated out of IBA1+ cells.

### Quantification and statistical analysis

Statistical analyses were performed with GraphPad Prism (version 8.4.2) and are specified in the figure legends. Paired t-tests were used to analyze statistical differences between the means of tissue before (‘native’) and after decellularization. Student’s t-tests were used to analyze statistical differences between two sample groups. When relative values were compared to control, untreated or glass conditions, set to 1 in each independent cell culture experiment, statistical significance was analyzed with a one-sample *t* test. One-way ANOVA followed by a Tukey’s multiple comparisons test was used to test for statistical differences between multiple tissue groups. Significant differences were visualized using ∗ (*p* < 0.05), and ∗∗ (*p* < 0.01). Exact *p* values and number of biological units are provided in Table S9.

## Supplemental information

Document S1. Figures S1–S8 and Table S1, S3, S5, S7, and S9

Table S2. Proteins detected in dCGM and dNAGM by LC-mass spectrometry

Table S4. Proteins detected in dCtrl, dDemyelinating, and dDemyelinated cortex by LC-mass spectrometry

Table S6. Proteins detected in dDemyelinated, dRemyelinating, and dRemyelinated cortex by LC-mass spectrometry

Table S8. Proteins detected in dPLGM, dGML low, and dGML high by LC-mass spectrometry
