# Supplementary material for: Pathological extracellular matrix changes in decellularized normal appearing gray matter and subpial multiple sclerosis lesions
Source: iScience. 2026 Jun 6;29(6):116020. doi: 10.1016/j.isci.2026.116020 (PMC13264259; doi:10.1016/j.isci.2026.116020)
Supplement: Document S1. Figures S1–S8 and Table S1, S3, S5, S7, and S9 [file mmc1.pdf]

iScience, Volume *29*

## Supplemental information

# Pathological extracellular matrix changes

# in decellularized normal appearing gray matter

# and subpial multiple sclerosis lesions

**Jody M. de Jong, Justina C. Wolters, Marion H.C. Wijering, Joop de Vries, Susanne M. Kooistra, Bart J.L. Eggen, and Wia Baron**

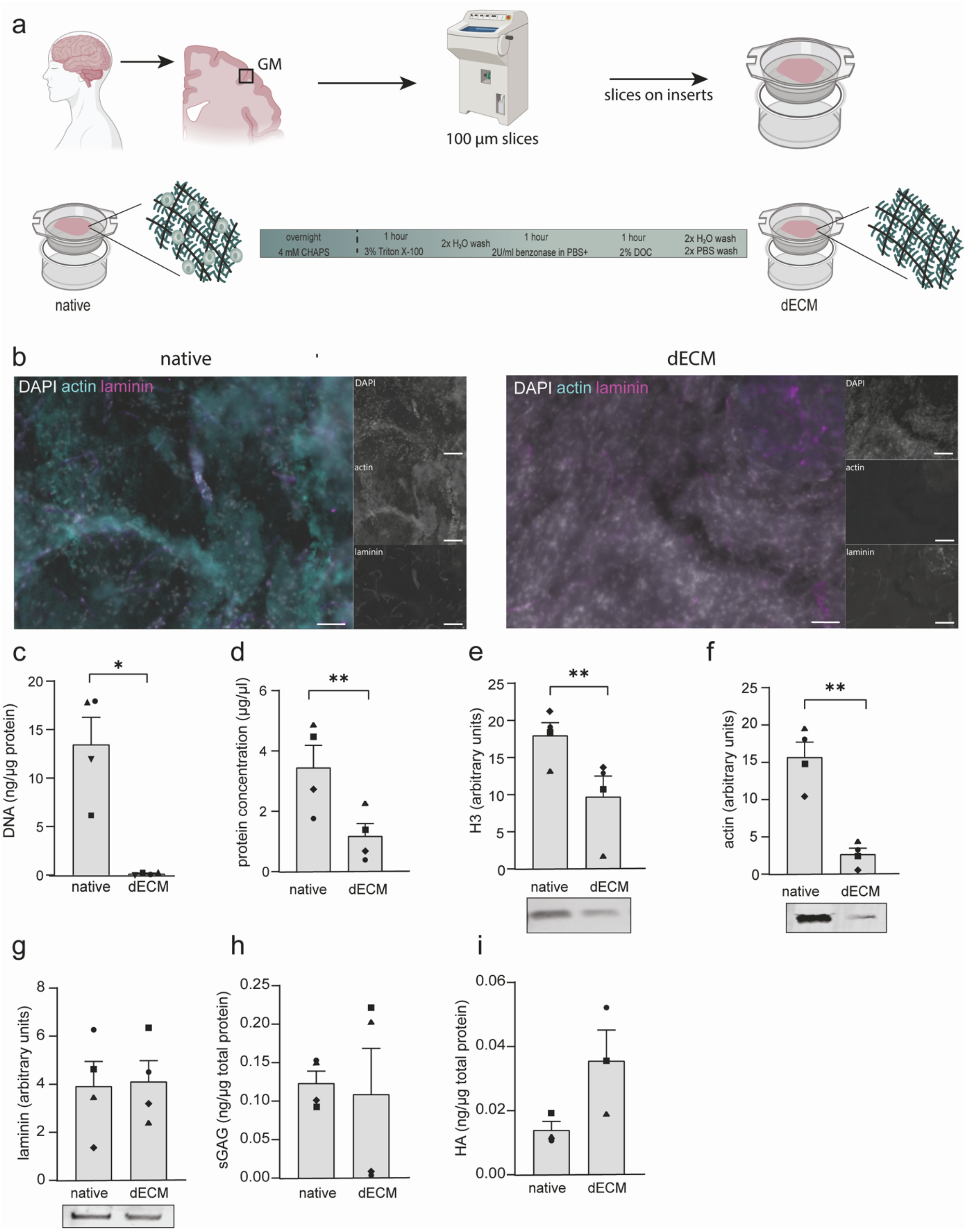


**Figure S1. Effective decellularization of human gray matter brain slices.** (**a**) Schematic overview of the experimental set-up and decellularization protocol for 100 µm snap-frozen frozen gray matter brain slices (NAGM, n=3-4). (**b**) Immunohistochemistry of actin (cyan) and laminin (magenta) on native and decellularized (dECM) slices. Nuclei were visualized with DAPI (white). Scale bars are 20 µm (overview) and 40 µm (inset). (**c**) DNA content of total protein of native and decellularized slices. (**d**) Protein concentration of native and decellularized slices. (**e-g**) Western blot quantification of nuclear protein histone H3 (**e**), cytoskeletal protein actin (**f**) and ECM protein laminin (**g**) of native and decellularized slices. (**h-i**) Quantification of ECM components sulfated glycosaminoglycans (sGAG, **h**) and hyaluronan (HA, **i**). Representative images (**b**) or blots (**e-g**) of at least three different donors are shown. Bars represent mean values. Error bars represent standard error of the mean. Symbols indicate different donors (**c-i**), and for each donor native and decellularized tissue were consecutive slices. Statistical analyses were performed using a paired student t-test to test for differences between native and decellularized slices (**c-i**, * $p<0.05$, ** $p<0.01$).

a

| GO-biological process in dCGM and dNAGM | p value |
|---|---|
| 1. neurofilament bundle assembly | 8.77E-03 |
| 2. 1-phosphatidyl-1D-mo-inositol 4,5-biphosphate biosynthetic process | 8.76E-03 |
| 3. positive regulation of platelet-derived growth factor receptor-beta signaling pathway | 8.75E-03 |
| 4. protein localization to juxtaparanode region of axon | 1.02E-03 |
| 5. isocitrate metabolic process | 1.13E-04 |
| 6. dendritic transport of messenger ribonucleoprotein complex | 8.73E-03 |
| 7. regulation of Golgi inheritance | 1.02E-03 |
| 8. postsynaptic intermediate filament cytoskeleton organization | 8.72E-03 |
| 9. regulation of clathrin coat assembly | 8.71E-03 |
| 10. SNARE complex disassembly | 8.71E-03 |

b

| GO-biological process in dCuprizone | p value |
|---|---|
| 1. positive regulation of calcium ion-dependent exocytosis of neurotransmitter | 3.97E-04 |
| 2. mesenchymal stem cell differentiation | 3.97E-04 |
| 3. glycerol-3-phosphate catabolic process | 5.41E-03 |
| 4. neurofilament bundle assembly | 3.97E-04 |
| 5. olfactory nerve formation | 5.41E-03 |
| 6. positive regulation of barbed-end actin filament capping | 5.41E-03 |
| 7. ITP biosynthetic process | 5.41E-03 |
| 8. cellular response to fluoride | 5.41E-03 |
| 9. positive regulation of protein lipidation | 5.41E-03 |
| 10. methylglyoxal biosynthetic process | 5.41E-03 |

c

| GO-biological process in dGML and dPLGM | p value |
|---|---|
| 1. neurofilament bundle assembly | 1.58E-02 |
| 2. adenine metabolic process | 1.58E-02 |
| 3. 1-phosphatidyl-1D-myo-inositol 4,5-bisphosphate biosynthetic process | 1.58E-02 |
| 4. positive regulation of platelet-derived growth factor receptor-beta signaling pathway | 1.58E-02 |
| 5. endoplasmic reticulum tubular network membrane organization | 1.56E-02 |
| 6. protein localization to juxtaparanode region of axon | 2.41E-03 |
| 7. regulation of translation at postsynapse, modulating synaptic transmission | 1.58E-02 |
| 8. regulation of endocannabinoid signaling pathway | 1.58E-02 |
| 9. dendritic transport of messenger ribonucleoprotein complex | 1.58E-02 |
| 10. microtubule cytoskeleton organization involved in establishment of planar polarity | 1.57E-02 |

**Figure S2. Gene Ontology (GO) terms associated with all proteins detected in decellularized gray matter brain tissue slices.** Top-10 GO-terms related to biological process enriched in decellularized control gray matter (dCGM) and normal appearing gray matter (dNAGM) (**a**), decellularized cortex of control, 3 weeks and 5 weeks cuprizone-fed mice (dCuprizone) (**b**) and decellularized subpial gray matter lesions (dGML) and perilesional gray matter (dPLGM) (**c**) are indicated.

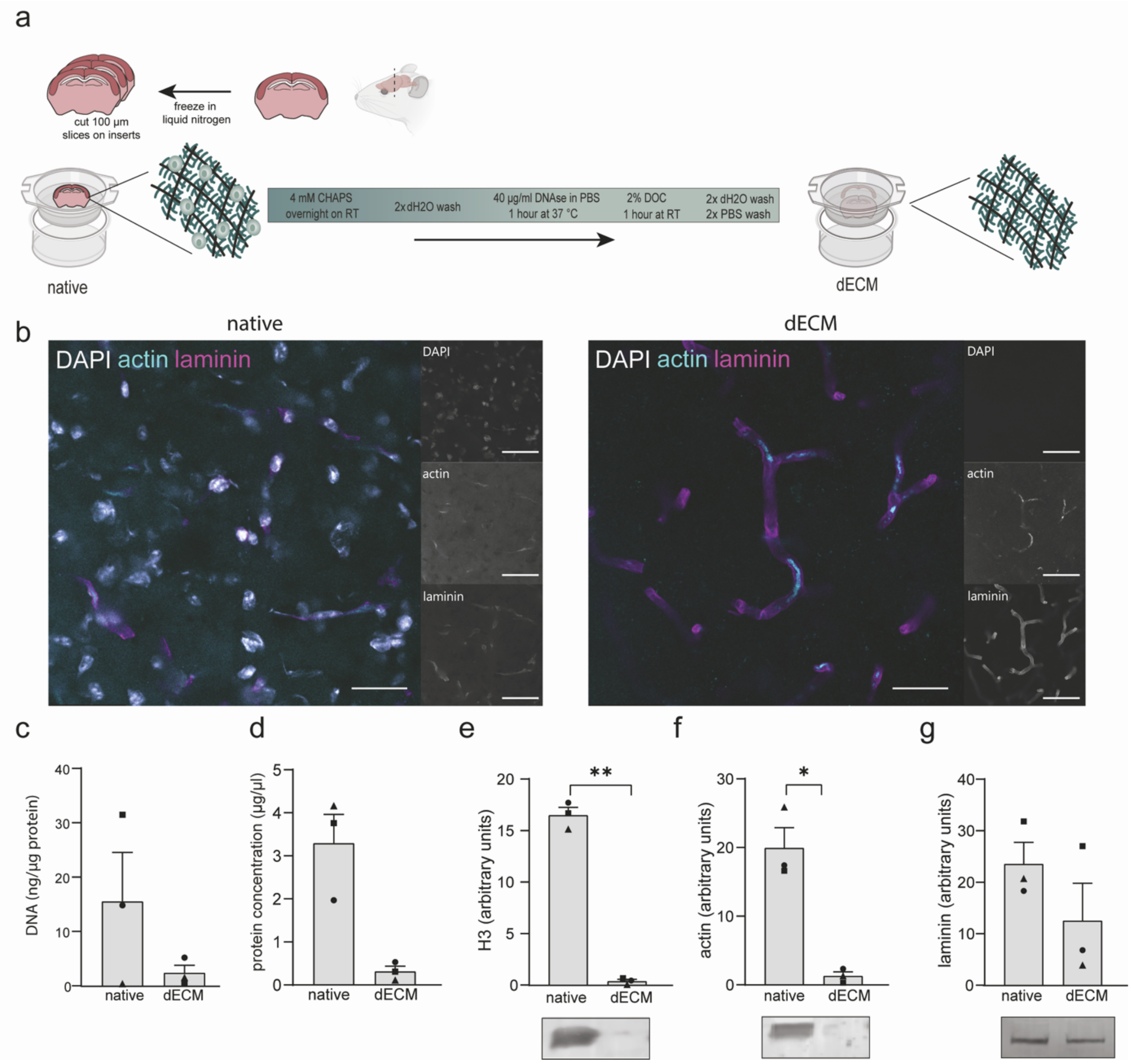


**Figure S3. Effective decellularization of thin mouse cortex slices.** (**a**) Schematic overview of the experimental set-up and decellularization protocol for 100 µm snap-frozen mouse brain slices (n=3). (**b**) Immunohistochemistry of actin (cyan) and laminin (magenta) on native and decellularized (dECM) mouse slices. Nuclei were visualized with DAPI (white). Scale bars are 20 µm (overview) and 40 µm (inset). (**c**) DNA content of total protein of native and decellularized slices. (**d**) Protein concentration of native and decellularized slices. (**e-g**) Western blot quantification of nuclear protein histone H3 (**e**), cytoskeletal protein actin (**f**) and ECM protein laminin (**g**) of native and decellularized slices. Representative images (**b**) or blots (**e-g**) of three independent experiments are shown. In each independent experiment native and decellularized mouse brain tissue were consecutive slices. Bars represent mean values. Error bars represent standard error of the mean. Symbols represent independent decellularization experiments. Statistical analyses were performed using a student paired t-test to test for differences between native and decellularized slices (**c-g**, * $p<0.05$, ** $p<0.01$).

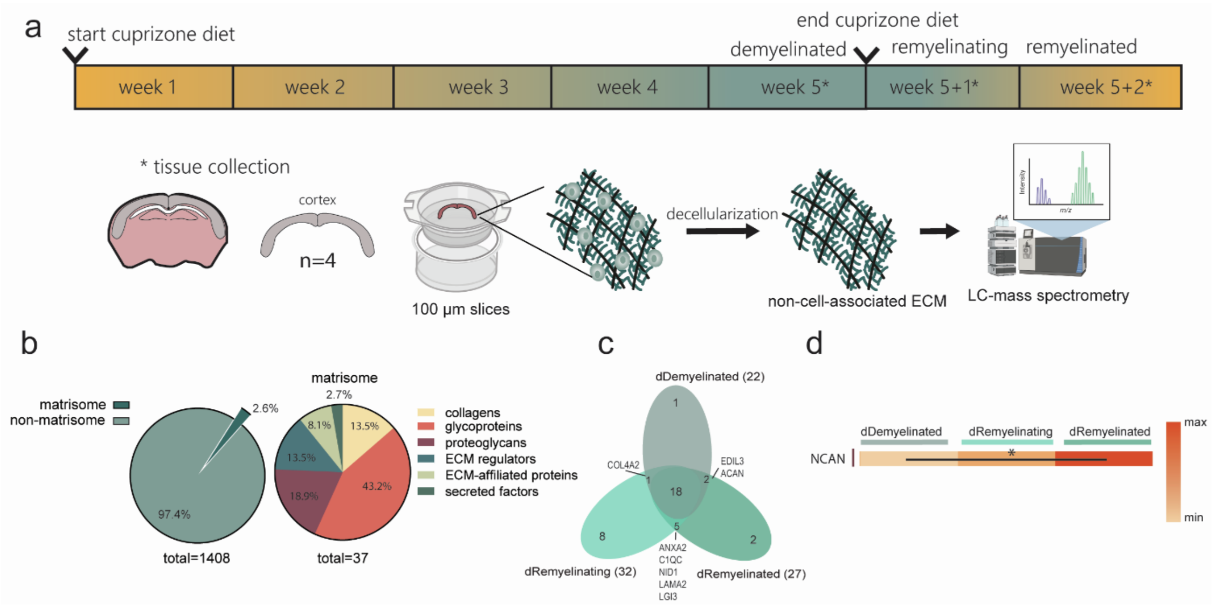


**Figure S4. NCAN is more abundant at 2 weeks cuprizone withdrawal.** (**a**) Experimental set-up. Mice were fed with cuprizone for 5 weeks (demyelinat*ed*, n=4), 5 weeks cuprizone followed by 1 week normal chow (remyelinat*ing*, n=4) or 2 weeks normal chow (remyelinat*ed*, n=4). Fresh frozen cortices were sectioned in 100 µm slices and decellularized on inserts. Proteins in the decellularized tissue slices were identified with LC-mass spectrometry and analysed with label free quantitative proteomics. (**b**) Pie charts depicting the distribution of matrisome and non-matrisome proteins in decellularized demyelinated (dDemyelinated), remyelinating (dRemyelinating) and remyelinated (dRemyelinated) cortex (left pie chart) and the distribution of different matrisome proteins over the indicated ECM-core and ECM-associated groups (right pie chart). (**c**) Venn diagram depicting the number of unique and common matrisome proteins in dDemyelinated, dRemyelinating and dRemyelinated. In parentheses the number of detected matrisome proteins present in at least one sample are indicated. Unique matrisome proteins found in at least two out of four samples in one group or in at least one sample of two groups are indicated. (**d**) Heatmap showing the relative abundance of the differentially abundant matrisome proteins. Statistical analyses were performed using a student's t-test to test for differentially abundant matrisome proteins between dDemyelinated and dRemyelinating, dDemyelinated and dRemyelinated, and dRemyelinating and dRemyelinated (**d**, *$p<0.05$).

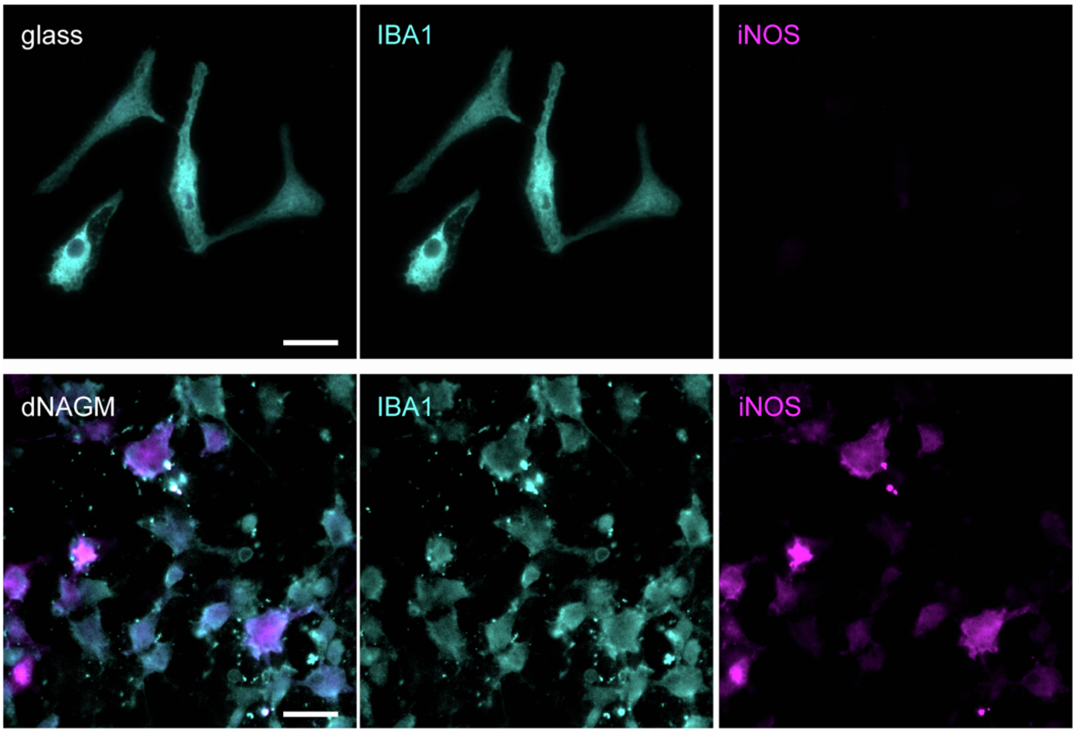


**Figure S5. iNOS/IBA1-stained primary microglia cultured on glass or introduced to decellularized NAGM.** Primary microglia were cultured on uncoated glass or introduced to 100 um slices of decellularized normal appearing gray matter (dNAGM). Microglia are co-stained for microglia marker IBA1 (cyan) and pro-inflammatory iNOS (magenta). Representative high magnification images visualizing IBA1+ microglia morphology and iNOS immunoreactivity are shown. Scale bars are 25 μm.

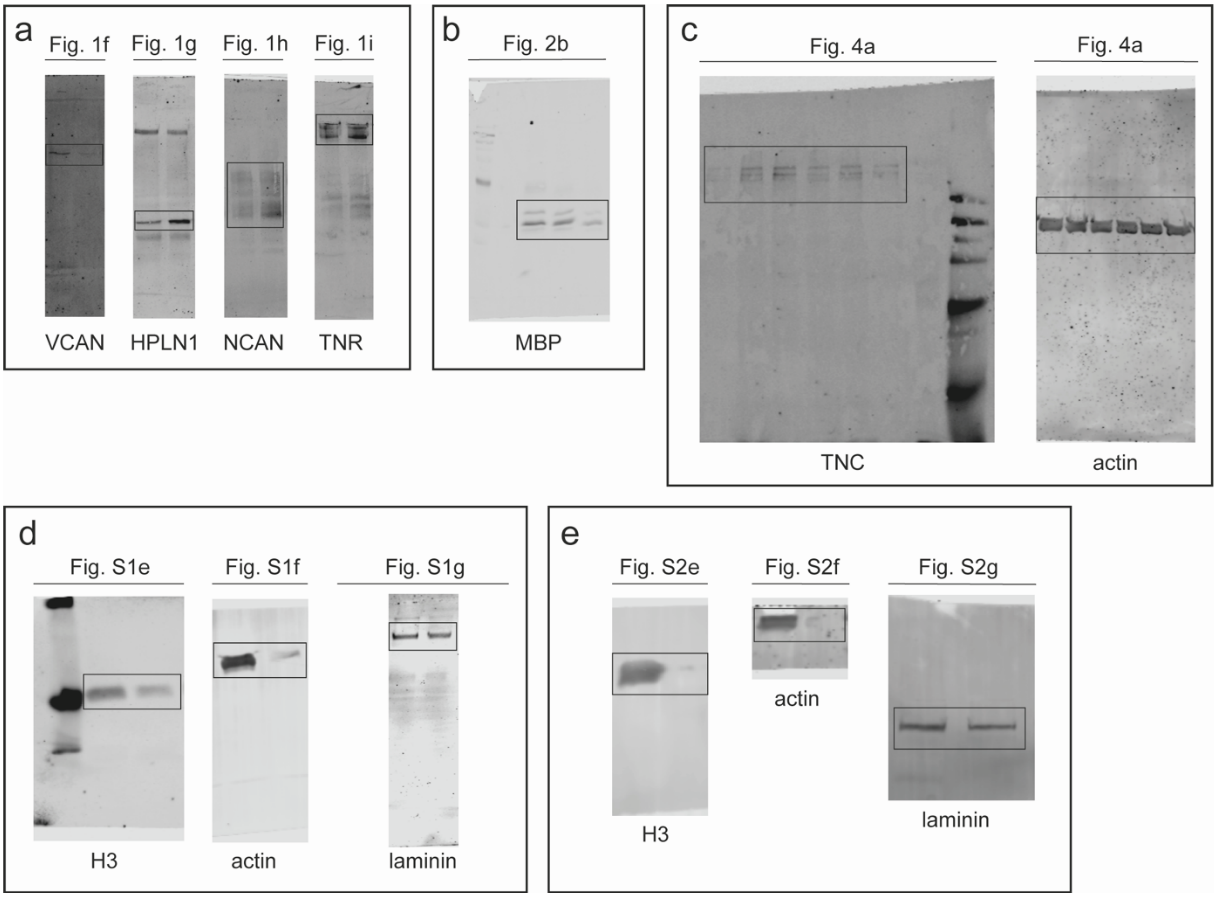


**Figure S6. Full blot immunoblot scans.** Full scans of the indicated immunoblots in figure 1 (**a**), figure 2 (**b**), figure 4 (**c**), supplementary figure 1 **(d)** and supplementary figure 2 (**e**). The boxed areas are presented in the respective figures. Following transfer, some membranes were cut horizontally due to the limited amount of sample; therefore, only the parts corresponding to the appropriate molecular weight of the protein of interest were incubated with the antibody. Notably, the immunoblot for HPLN1 (**a**) was first incubated with an anti-laminin antibody (upper band).

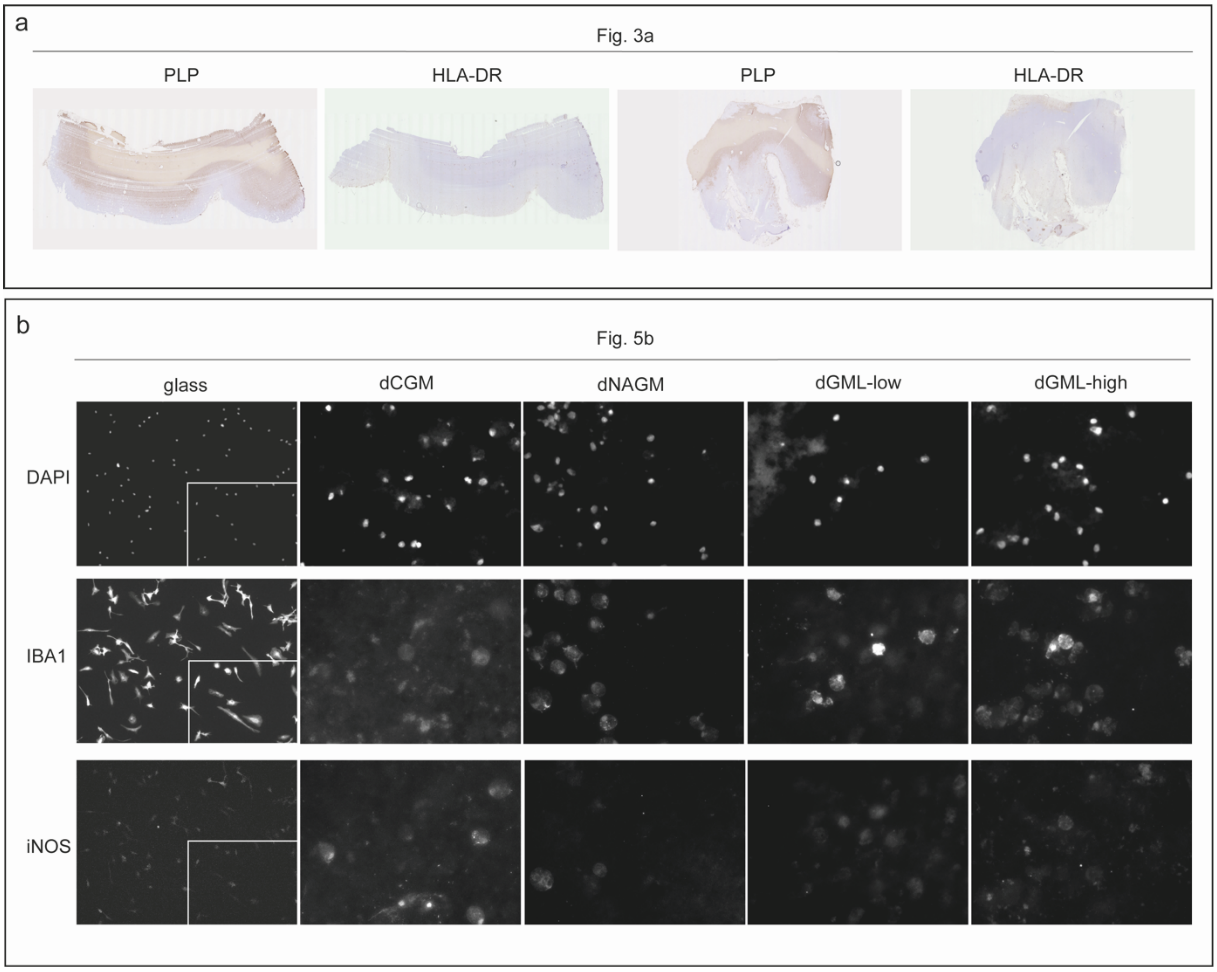


**Figure S7. Original microscopy images.** Original microscopy images of the indicated images presented in figure 3 (**a**), and figure 5 (**b**), The boxed areas are presented in the respective figures.

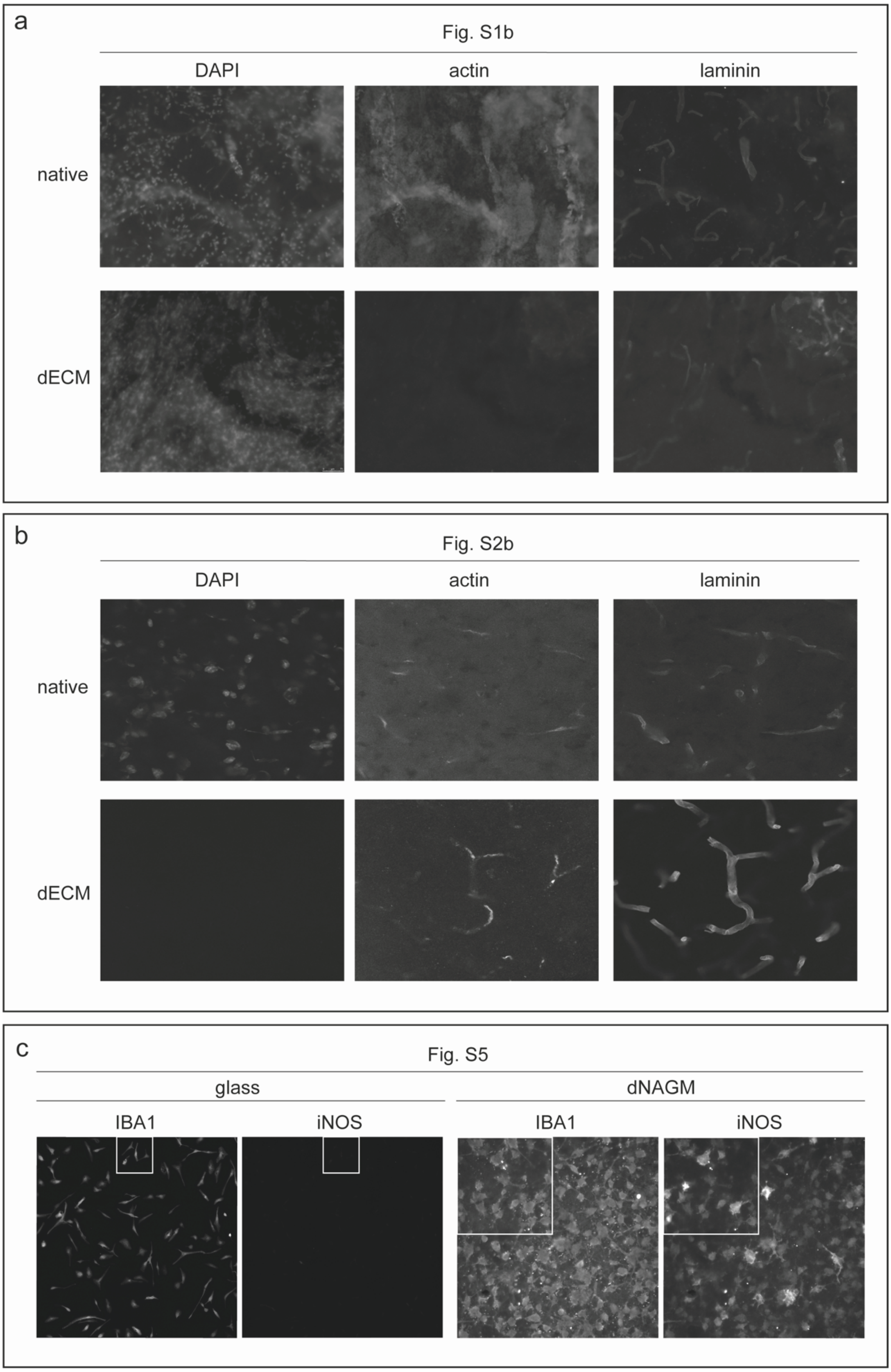


**Figure S8. Original microscopy images.** Original microscopy images of the indicated images presented in figure S1 (**a**), figure S2 (**b**) and figure S5 (**c**). The boxed areas are presented in the respective figures.

**Table S1.** *ECM proteins detected in decellularized CGM and NAGM by LC-mass spectrometry divided in matrisome groups*

| | dCGM | | | | dNAGM | | | |
|---|---|---|---|---|---|---|---|---|
| donor | 1 ▲ | 2 ■ | 3 ◆ | 4 ● | 1 ▲ | 2 ■ | 3 ◆ | 4 ● |
| DIFFERENTIAL PROTEINS[a] | | | | | | | | |
| TNR | 33.76 | 33.47 | 34.07 | 33.64 | 32.76 | 32.21 | 31.72 | 33.19 |
| NCAN | 28.51 | 28.77 | 30.30 | 28.39 | 32.44 | 31.39 | 30.67 | 31.22 |
| VCAN | 36.87 | 36.26 | 36.72 | 35.77 | 35.18 | 34.81 | 33.07 | 35.68 |
| HPLN1 | 28.77 | 28.86 | 29.13 | | 32.24 | 31.61 | 30.27 | 30.58 |
| UNIQUE PROTEINS in dCGM[b] | | | | | | | | |
| FGA | 28.56 | | | | | | | |
| FGB | 30.45 | | | | | | | |
| FGG | 30.88 | | | | | | | |
| CSTB | 26.14 | | | | | | | |
| SERPINH1 | 26.42 | | | | | | | |
| ANXA1 | 29.49 | | | | | | | |
| LGALS1 | 28.07 | | | | | | | |
| SEMA7A | | | 25.20 | | | | | |
| ANXA5 | 30.51 | 28.25 | 27.18 | | | | | |
| S100A1 | | | 26.14 | | | | | |
| UNIQUE PROTEINS in dNAGM[b] | | | | | | | | |
| AGRN | | | | | | 26.73 | | |
| LAMB2 | | | | | 27.32 | | 27.42 | |
| LGI1 | | | | | 28.09 | 27.55 | | 27.89 |
| TINAGL1 | | | | | 28.24 | 29.97 | 28.79 | 26.45 |
| HSPG2 | | | | | 26.63 | 27.44 | | |
| HPLN4 | | | | | 28.58 | | 28.94 | 28.88 |
| ADAM22 | | | | | 27.26 | | 27.18 | |
| CTSB | | | | | 25.52 | | | |
| PLXNA4 | | | | | 28.86 | 26.89 | 26.34 | 27.30 |
| C1QB | | | | | 27.04 | | | |
| COMMON PROTEINS[c] | | | | | | | | |
| TNC | 30.21 | 27.06 | 30.22 | 30.46 | | | | 28.44 |
| LGI3 | 28.54 | 28.89 | 28.30 | 29.46 | 28.21 | 28.83 | 29.51 | 29.11 |
| HPLN2 | 34.13 | 34.22 | 35.47 | 34.41 | 34.27 | 33.83 | 32.96 | 33.72 |
| ACAN | 28.16 | 28.22 | 30.41 | 27.81 | 28.21 | 27.09 | | 27.56 |
| BCAN | 30.95 | 29.10 | 31.03 | 27.80 | 30.23 | 28.76 | | 30.65 |
| ADAM23 | | | 27.60 | | 27.50 | 26.88 | | |
| ANXA6 | 31.91 | 31.07 | 30.93 | 28.37 | 29.77 | 30.24 | 29.92 | 30.03 |

[a]Differential proteins are differentially abundant between dNAGM and dCGM. [b]Unique proteins are present in a single tissue group. [c]Common proteins are equally abundant across tissue groups. dNAGM, decellularized normal appearing gray matter; dCGM, decellularized control gray matter. Colours indicate matrisome groups with orange = ECM glycoproteins; dark red = proteoglycans; teal = ECM regulators; light green = ECM-affiliated proteins; dark green = secreted factors; symbols represent different donors (**table 1**).

**Table S3.** *ECM proteins detected in decellularized cortex in the dietary-cuprizone mouse model by LC-mass spectrometry divided in matrisome groups*

| | dCtrl | | | | dDemyelinating | | | | dDemyelinated | | | |
|---|---|---|---|---|---|---|---|---|---|---|---|---|
| animal | 1 | 2 | 3 | 4 | 1 | 2 | 3 | 4 | 1 | 2 | 3 | 4 |
| DIFFERENTIAL PROTEINS[a] | | | | | | | | | | | | |
| NCAN | 77.20 | 34.10 | 50.40 | 60.50 | 59.20 | 52.70 | 33.90 | 46.40 | 32.30 | 19.00 | 33.70 | 20.30 |
| HPLN1 | 74.00 | 48.30 | 53.30 | 73.00 | 62.90 | 35.80 | 39.60 | 43.40 | 28.10 | 19.60 | 39.60 | 23.00 |
| UNIQUE PROTEINS in dDemyelinating[b] | | | | | | | | | | | | |
| TNC | | | | | | | 1.21 | | | | | |
| ELFN2 | | | | | | | 0.57 | | | | | |
| PLXNA1 | | | | | | 2.63 | | | | | | |
| PLXNA3 | | | | | | | 0.06 | | | | | |
| PLXND1 | | | | | | | 0.94 | | | | | |
| ADAM11 | | | | | | | | 0.73 | | | | |
| S100A16 | | | | | | 1.71 | 0.24 | | | | | |
| UNIQUE PROTEINS in dDemyelinated[b] | | | | | | | | | | | | |
| COL4A2 | | | | | | | | | | 3.09 | | |
| NTNG2 | | | | | | | | | | | 0.81 | |
| UNIQUE PROTEINS in dCtrl[b] | | | | | | | | | | | | |
| COL1A1 | 0.39 | | | | | | | | | | | |
| LGI3 | | 1.76 | | | | | | | | | | |
| GPC1 | | | 1.67 | | | | | | | | | |
| UNIQUE PROTEINS in dCtrl and dDemyelinating[b] | | | | | | | | | | | | |
| LAMA2 | | | 1.79 | | | | | 1.95 | | | | |
| ANXA2 | | | 2.93 | | 0.87 | 1.43 | | | | | | |
| C1QC | 6.35 | | 2.62 | | | | 1.71 | 7.97 | | | | |
| COMMON PROTEINS[c] | | | | | | | | | | | | |
| AGRN | 0.73 | | 1.20 | | 1.34 | 3.70 | | 4.24 | 2.93 | | 0.81 | |
| LAMC1 | 6.26 | | 8.56 | 1.90 | 3.64 | 12.80 | 1.48 | 16.50 | 5.47 | 0.89 | 2.28 | |
| LAMB2 | 10.80 | | 13.00 | | 3.94 | 19.30 | 1.21 | 24.00 | 9.23 | | 4.03 | |
| LGI1 | 9.51 | 27.00 | 14.30 | 19.40 | 20.20 | 5.98 | 15.40 | 10.20 | 12.70 | 8.51 | 26.90 | 19.60 |
| ACAN | 1.12 | | | | 1.56 | | 0.66 | | 0.79 | | | |
| BCAN | 21.30 | 2.89 | 32.40 | 5.08 | 10.40 | 15.10 | 2.65 | 15.10 | 12.50 | | 13.90 | |
| HPLN4 | | 4.75 | | 4.68 | | | 2.52 | 3.74 | 2.79 | 1.67 | 4.42 | 3.65 |
| HSPG2 | | | 4.65 | | 7.7 | 1.34 | | 7.3 | 1.16 | | | |
| VCAN | 4.18 | 2.85 | 2.74 | 4.61 | 2.20 | 1.82 | 1.95 | 5.03 | 2.52 | | 1.58 | |
| ANXA6 | | 2.66 | | | | | 1.12 | 0.95 | 1.51 | | | 1.63 |
| C1QB | 7.88 | | 2.93 | 3.40 | | 2.93 | 3.88 | 16.10 | | | 5.70 | |
| PLXNA4 | | 4.93 | | 1.82 | 1.53 | | 5.10 | 0.91 | | 1.90 | 1.22 | 1.19 |
| ADAM22 | 3.80 | 2.60 | | | 2.81 | 4.22 | 2.74 | 14.40 | 5.85 | | 3.48 | 3.90 |
| ADAM23 | 1.47 | 1.26 | 2.62 | | | 3.06 | 0.96 | 4.07 | 1.29 | | 2.23 | 2.43 |

[a] Differential proteins are differentially abundant between two or more tissue groups. [b]Unique proteins are present in a single tissue group. [c]Common proteins are equally abundant across groups. dCtrl - decellularized control cortex, dDemyelinating - decellularized cortex after 3 weeks cuprizone feeding, dDemyelinated - decellularized cortex after 5 weeks cuprizone feeding. Colours indicate matrisome groups with orange = ECM glycoproteins; dark red = proteoglycans; teal = ECM regulators; light green = ECM-affiliated proteins; dark green = secreted factors.

**Table S5.** *ECM proteins detected in decellularized cortex in the dietary cuprizone mouse model by LC-mass spectrometry divided in matrisome groups.*

| | dDemyelinated | | | | dRemyelinating | | | | dRemyelinated | | | |
|---|---|---|---|---|---|---|---|---|---|---|---|---|
| animal | 1 | 2 | 3 | 4 | 1 | 2 | 3 | 4 | 1 | 2 | 3 | 4 |
| DIFFERENTIAL PROTEINS[a] | | | | | | | | | | | | |
| NCAN | 32.25 | 18.99 | 33.73 | 20.33 | 24.99 | 54.81 | 38.13 | 55,54 | 124.2 | 60.65 | 73.29 | 36.5 |
| UNIQUE PROTEINS in dDemyelinated[b] | | | | | | | | | | | | |
| NTNG2 | | | 0.81 | | | | | | | | | |
| UNIQUE PROTEINS in dRemyelinating[b] | | | | | | | | | | | | |
| COL1A2 | | | | | | 0.97 | | | | | | |
| COL6A1 | | | | | 0.59 | | | | | | | |
| ELN | | | | | 1.26 | | | | | | | |
| LAMA5 | | | | | 0.93 | | | | | | | |
| VWA1 | | | | | | 0.38 | | | | | | |
| TINAGL1 | | | | | 6.68 | | | | | | | |
| BRINP2 | | | | | 0.43 | | | | | | | |
| TGM2 | | | | | 0.34 | | | | | | | |
| UNIQUE PROTEINS in dRemyelinated[b] | | | | | | | | | | | | |
| COL12A1 | | | | | | | | | | | 2.96 | |
| LAMB1 | | | | | | | | | | | 0.42 | |
| UNIQUE PROTEINS in dDemyelinated and dRemyelinated[b] | | | | | | | | | | | | |
| ACAN | 0.79 | | | | | | | | | | 1.56 | 1.04 |
| EDIL3 | 3.11 | | | | | | | | | | 4.56 | |
| UNIQUE PROTEINS in dDemyelinated and dRemyelinating[b] | | | | | | | | | | | | |
| COL4A2 | | 3.09 | | | 2.71 | | | | | | | |
| UNIQUE PROTEINS in dRemyelinating and dRemyelinated[b] | | | | | | | | | | | | |
| NID1 | | | | | | 2.03 | | | | | 3.19 | |
| LAMA2 | | | | | | 1.98 | | | | | 4.51 | |
| LGI3 | | | | | 0.69 | | | | | | | 1.29 |
| ANXA2 | | | | | | 1.01 | | | | 1.19 | | |
| C1QC | | | | | | 2.13 | | | | | 10.59 | |
| COMMON PROTEINS[c] | | | | | | | | | | | | |
| COL4A1 | | 4.98 | | | 9.48 | | | | | | | 2.67 |
| HSPG2 | 1.16 | | | | 6.84 | 4.99 | | | 2.14 | | 5.57 | |
| BCAN | 12.48 | | 13.92 | | 6.51 | 20.66 | 15.87 | 26.83 | 79.55 | 17.15 | 52.67 | |
| VCAN | 2.52 | | 1.58 | | 2.31 | 3.67 | | | 5.45 | | 3.65 | |
| HPLN4 | 2.79 | 1.66 | 4.42 | 3.64 | 6.09 | | | 2.11 | | | | 5.40 |
| HPLN1 | 28.10 | 19.60 | 39.57 | 23.01 | 36.10 | 49.33 | 23.69 | 58.64 | 84.59 | 34.55 | 74,00 | 34.80 |
| AGRN | 2.93 | | 0.81 | | | 2.38 | 1.05 | | 1.33 | | 12.97 | |
| LAMC1 | 5.47 | 0.89 | 2.28 | | 1.27 | 14.72 | 1.89 | 5.26 | 3.91 | 3.42 | 26.60 | 0.40 |
| LAMA1 | 1.15 | | | | | 0.78 | | | | | 10.30 | |

| | | | | | | | | | | | | |
|---|---|---|---|---|---|---|---|---|---|---|---|---|
| LAMB2 | 9.22 | | 4.03 | | 3.85 | 15.42 | 1.89 | 5.41 | 9.90 | 9.69 | 38.60 | 2.13 |
| TNR | 30.34 | 25.24 | 33.93 | 39.09 | 35.56 | 42.46 | 36.20 | 41.27 | 64.28 | 32.37 | 42.72 | 40.64 |
| LGI1 | 12.72 | 8.51 | 26.89 | 19.61 | 27.57 | 13.15 | | | 1.34 | | 2.82 | 25.21 |
| C1QB | | | 5.70 | | | 3.31 | | | 7.79 | | 13.23 | |
| ANXA6 | 1.51 | | | 1.63 | 1.07 | | | | | | 1.04 | 1.64 |
| PLXNA4 | | 1.90 | 1.22 | 1.19 | 5.93 | 0.71 | | | | | | 2.46 |
| ADAM22 | 5.84 | | 3.48 | 3.90 | 2.85 | 5.31 | 1.96 | 5.90 | 2.46 | | 9.62 | |
| ADAM23 | 1.29 | | 2.23 | 2.43 | 2.17 | | | | | | 7.32 | |

[a] Differential proteins are differentially abundant between two or more tissue groups. [b]Unique proteins are present in a single tissue group. [c]Common proteins are equally abundant across groups. dDemyelinated - decellularized cortex after 5 weeks cuprizone feeding, dRemyelinating – decellularized cortex after 5 weeks cuprizone feeding and 1 week normal chow, dRemyelinated - decellularized cortex after 5 weeks cuprizone feeding and 2 weeks normal chow. Colours indicate matrisome groups with orange = ECM glycoproteins; dark red = proteoglycans; teal = ECM regulators; light green = ECM-affiliated proteins; dark green = secreted factors.

**Table S7.** *ECM proteins detected in decellularized GML and PLGM by LC-mass spectrometry divided in matrisome groups*

| | dPLGM | | | | dGML-low | | | | dGML-high | | | |
|---|---|---|---|---|---|---|---|---|---|---|---|---|
| donor | 1 ● | 2 ▲ | 3 ■ | 4 ◆ | 1 ● | 2 ▲ | 3 ■ | 4 ◆ | 1 ● | 2 ▲ | 3 ■ | 4 ◆ |
| DIFFERENTIAL PROTEINS[a] | | | | | | | | | | | | |
| TNC | 23.10 | | 22.36 | 23.17 | | 25.00 | 22.01 | 21.10 | 20.61 | | 20.97 | |
| UNIQUE PROTEINS in dPLGM[b] | | | | | | | | | | | | |
| LAMC1 | | | | 22.67 | | | | | | | | |
| LAMA2 | | | | 20.94 | | | | | | | | |
| NID2 | | | | 20.45 | | | | | | | | |
| UNIQUE PROTEINS in both dGML[b] | | | | | | | | | | | | |
| EDIL3 | | | | | 23.08 | 22.56 | 23.70 | | | | 22.78 | |
| FGB | | | | | 25.41 | | 23.26 | 24.07 | 23.47 | | 25.36 | |
| BGN | | | | | 22.69 | | | | | | 24.81 | |
| CSTB | | | | | | | 21.82 | | 21.33 | | 21.50 | |
| UNIQUE PROTEINS in dGML-low[b] | | | | | | | | | | | | |
| CILP | | | | | | | | 21.05 | | | | |
| CATB | | | | | | | | 20.12 | | | | |
| PLXNB1 | | | | | | 20.71 | | | | | | |
| PLXNC1 | | | | | | 22.19 | | | | | | |
| PLXND1 | | | | | | | | 20.47 | | | | |
| S100A8 | | | | | 23.31 | 21.38 | | | | | | |
| CRTAC1 | | | | | | | | 21.30 | | | | |
| UNIQUE PROTEINS in dGML-high[b] | | | | | | | | | | | | |
| COL18A1 | | | | | | | | | | | 21.81 | |
| COL14A1 | | | | | | | | | | | 24.69 | |
| COL12A1 | | | | | | | | | | | 24.88 | |
| LAMA5 | | | | | | | | | | | 19.10 | |
| CST3 | | | | | | | | | | | 21.63 | |
| CTSD | | | | | | | | | | | 22.38 | |
| C1QB | | | | | | | | | | | 22.00 | |
| C1QC | | | | | | | | | | | 21.75 | |
| S100A1 | | | | | | | | | | 20.05 | | |
| COMMON PROTEINS[c] | | | | | | | | | | | | |
| COL4A1 | | | | 25.89 | | 22.22 | | 24.91 | 22.40 | | | |
| COL4A2 | | | | 24.68 | | | | 22.80 | 22.32 | | | |
| COL6A1 | | | | 24.75 | 24.06 | 22.33 | | 22.39 | 22.62 | 25.58 | 24.16 | 23.09 |
| COL6A3 | 22.85 | 24.07 | 21.90 | 25.64 | 24.43 | 22.11 | | 23.01 | 22.55 | 25.27 | 24.98 | 24.81 |
| AGRN | | | | 23.10 | 23.39 | 22.68 | 22.49 | 22.96 | 22.40 | | 24.26 | |
| LGI1 | | 24.65 | 23.48 | 24.76 | 24.43 | 23.32 | 23.28 | 24.20 | 24.03 | | 23.99 | 24.06 |
| FGA | | | 24.59 | 22.59 | 25.21 | 21.88 | 24.05 | 24.51 | 22.79 | | 26.55 | |
| FGG | | | 24.90 | | 25.65 | | 24.23 | 24.86 | 24.10 | | 25.52 | |
| LAMB2 | | | | 23.37 | | | | 23.15 | | | | 22.63 |
| LGI4 | | | | 22.97 | | | | 22.52 | 22.54 | | | |
| LGI3 | | | 24.93 | 25.79 | 24.36 | 23.80 | 23.39 | 25.45 | 24.54 | | 23.93 | |
| TNR | 29.52 | 29.17 | 29.18 | 29.38 | 29.46 | 29.46 | 28.69 | 28.58 | 28.94 | 29.66 | 29.53 | 29.26 |

| | | | | | | | | | | | | |
|---|---|---|---|---|---|---|---|---|---|---|---|---|
| TINAGL1 | 21.03 | 22.23 | 21.47 | 24.16 | 21.60 | 20.69 | 20.64 | 23.13 | 21.76 | | | 22.30 |
| NCAN | 28.85 | 28.53 | 27.82 | 28.05 | 29.29 | 28.21 | 27.83 | 27.72 | 27.95 | 28.40 | 28.93 | 28.85 |
| HPLN1 | 28.41 | 28.33 | 27.58 | 27.63 | 28.82 | 27.79 | 27.40 | 26.41 | 27.60 | 27.97 | 28.89 | 28.44 |
| VCAN | 31.17 | 29.58 | 29.73 | 30.33 | 29.08 | 30.57 | 29.98 | 29.39 | 29.21 | 30.02 | 29.28 | 28.74 |
| ACAN | | | 24.02 | 23.69 | 23.55 | 23.74 | 23.40 | 22.37 | 24.24 | | 24.68 | |
| HSPG2 | | | | 25.54 | 22.32 | 20.24 | | 23.67 | 21.67 | | | |
| HPLN4 | 24.52 | 24.63 | 25.24 | 25.71 | 25.27 | 24.16 | 24.53 | 25.19 | 24.59 | | 26.05 | 25.33 |
| BCAN | 23.94 | 23.43 | 24.81 | 25.71 | 25.28 | 25.02 | 25.06 | 25.14 | 24.46 | 24.62 | 26.21 | |
| HPLN2 | 30.73 | 28.70 | 29.58 | 30.43 | 29.22 | 29.28 | 28.90 | 28.76 | 29.04 | 29.49 | 29.57 | 28.70 |
| ADAM23 | | | | 22.57 | 22.82 | 22.71 | 22.94 | 23.05 | 23.05 | 22.51 | 23.41 | |
| SERPINA1 | | 22.49 | | | | | 20.55 | | 19.96 | | | |
| ADAM22 | | | | 23.93 | 23.64 | 23.72 | 24.58 | 23.49 | 23.88 | | 24.09 | 23.48 |
| C1QA | | | | 19.96 | | | 20.68 | | | | 23.14 | |
| ANXA2 | | | | 21.04 | 23.41 | 21.11 | | | | | 22.00 | 22.02 |
| ANXA6 | 26.63 | 28.04 | 28.15 | 28.15 | 28.25 | 26.81 | 27.30 | 27.00 | 27.09 | 27.81 | 27.51 | 28.18 |
| ANXA5 | | 21.90 | 27.29 | 22.93 | | 23.58 | 24.69 | 24.11 | 23.24 | 22.12 | 25.04 | |
| LEG1 | | | | 22.16 | | 21.68 | 21.98 | 21.70 | 22.17 | | | |
| LMAN1 | | | | 23.29 | | 23.36 | 23.72 | 23.43 | 23.68 | | | |
| PLXNA4 | | | 23.68 | 23.53 | 23.54 | 23.49 | 23.27 | 23.63 | 23.80 | | 23.34 | |
| PLXNA1 | 23.59 | | 25.14 | 24.91 | 24.69 | 24.84 | 25.61 | 24.77 | 24.83 | 24.46 | 24.70 | |
| S100B | 22.22 | | | 23.41 | 21.54 | | 21.53 | | 21.68 | | 22.23 | 21.45 |

[a]Differential proteins are differentially abundant between two or more tissue groups. [b]Unique proteins are absent in a single tissue group. [c]Common proteins are equally abundant across tissue groups. dGML-low, decellularized subpial gray matter lesion with low HLA-DR+ microglia number; dGML-high, decellularized subpial gray matter lesion with high HLA-DR+ microglia number, dPLGM, decellularized perilesional gray matter. Colours indicate matrisome groups with orange = ECM glycoproteins; dark red = proteoglycans; teal = ECM regulators; light green = ECM-affiliated proteins; dark green = secreted factors; symbols represent different donors (**table 1**).

**Table S9.** *Exact p values and number of biological units of the significant findings.*

| figure | p value | number of biological units |
|---|---|---|
| **1e** | | |
| TNR | 0.0103 | 8 |
| HPLN1 | 0.0092 | 8 |
| NCAN | 0.0056 | 8 |
| VCAN | 0.0322 | 8 |
| **1f** | 0.0125 | 8 |
| **1g** | 0.0256 | 8 |
| **2e** | | |
| HPLN1 dCTRL vs dDemyelinated | 0.0043 | 8 |
| NCAN dCTRL vs dDemyelinated | 0.0180 | 8 |
| HPLN1 dDemyelinating vs dDemyelinated | 0.0396 | 8 |
| NCAN dDemyelinating vs dDemyelinated | 0.0189 | 8 |
| **3d** | 0.0099 | 8 |
| **5c** | | 16 |
| dNAGM | 0.0154 | |
| dGML-low | 0.0087 | |
| dGML-high | 0.0028 | |
| **5d** | 0.0045 | 16 |
| **S1c** | 0.0180 | 8 |
| **S1d** | 0.0086 | 8 |
| **S1e** | 0.0055 | 8 |
| **S1f** | 0.0017 | 8 |
| **S3e** | 0.0015 | 6 |
| **S3f** | 0.0247 | 6 |
| **S4d** | 0.0167 | 8 |
